# A low FODMAP diet is associated with changes in the microbiota and reduction in breath hydrogen but not colonic volume in healthy subjects

**DOI:** 10.1371/journal.pone.0201410

**Published:** 2018-07-26

**Authors:** Tim J. Sloan, Jonna Jalanka, Giles A. D. Major, Shanthi Krishnasamy, Sue Pritchard, Salah Abdelrazig, Katri Korpela, Gulzar Singh, Claire Mulvenna, Caroline L. Hoad, Luca Marciani, David A. Barrett, Miranda C. E. Lomer, Willem M. de Vos, Penny A. Gowland, Robin C. Spiller

**Affiliations:** 1 The NIHR Nottingham Biomedical Research Centre (BRC) at Nottingham University Hospitals NHS Trust and the University of Nottingham, Nottingham, United Kingdom; 2 School of Life Sciences, University of Nottingham, Nottingham, United Kingdom; 3 Department of Clinical Microbiology, Nottingham University Hospitals NHS Trust, Nottingham, United Kingdom; 4 Nottingham Digestive Diseases Centre, School of Medicine, University of Nottingham, Nottingham, United Kingdom; 5 Immunobiology Research Program, University of Helsinki, Helsinki, Finland; 6 Sir Peter Mansfield Imaging Centre, School of Physics and Astronomy, University of Nottingham, Nottingham, United Kingdom; 7 Centre for Analytical Bioscience, Advanced Materials and Healthcare Technology Division, School of Pharmacy, University of Nottingham, Nottingham, United Kingdom; 8 Department of Nutritional Sciences, King’s College London, London, United Kingdom; University Hospital Llandough, UNITED KINGDOM

## Abstract

**Background & aims:**

Ingestion of poorly digested, fermentable carbohydrates (fermentable oligo-, di-, mono-saccharides and polyols; FODMAPs) have been implicated in exacerbating intestinal symptoms and the reduction of intake with symptom alleviation. Restricting FODMAP intake is believed to relieve colonic distension by reducing colonic fermentation but this has not been previously directly assessed. We performed a randomised controlled trial comparing the effect of a low FODMAP diet combined with either maltodextrin or oligofructose on colonic contents, metabolites and microbiota.

**Methods:**

A parallel randomised controlled trial in healthy adults (n = 37). All subjects followed a low FODMAP diet for a week and supplemented their diet with either maltodextrin (MD) or oligofructose (OF) 7g twice daily. Fasted assessments performed pre- and post-diet included MRI to assess colonic volume, breath testing for hydrogen and methane, and stool collection for microbiota analysis.

**Results:**

The low FODMAP diet was associated with a reduction in *Bifidobacterium* and breath hydrogen, which was reversed by oligofructose supplementation. The difference in breath hydrogen between groups post-intervention was 27ppm (95% CI 7 to 50, P<0.01). Colonic volume increased significantly from baseline in both groups (OF increased 110ml (19.6%), 95% CI 30ml to 190ml, P = 0.01; MD increased 90ml (15.5%), 95% CI 6ml to 175ml, P = 0.04) with no significant difference between them. Colonic volumes correlated with total breath hydrogen + methane. A divergence in *Clostridiales* abundance was observed with increased abundance of *Ruminococcaceae* in the maltodextrin group, while in the oligofructose group, *Lachnospiraceae* decreased. Subjects in either group with high methane production also tended to have high microbial diversity, high colonic volume and greater abundance of methanogens.

**Conclusion:**

A low FODMAP diet reduces total bacterial count and gas production with little effect on colonic volume.

## Introduction

Poorly digested, fermentable carbohydrates are believed to contribute to a healthy diet. Ingestion of ‘prebiotics’ that preferentially stimulate colonic bacteria[1], such as oligofructose and inulin, has been shown to reduce markers of metabolic syndrome while increasing the abundance of *Bifidobacteria*[2] and butyrate producers[3–5]. However intake of prebiotics may be limited by GI symptoms such as bloating, flatulence and abdominal discomfort[6]. These complex carbohydrates have been grouped as fermentable oligo-, di-, mono-saccharides and polyols (FODMAPs)[7] and dietary FODMAP restriction has been shown to relieve IBS symptoms[8–11]. A typical UK diet contains on average 29.6 g of FODMAPs the single largest component being fructose, either monomeric or as fructans, which averages 16.6 g / day while a typical patient following dietary advice for a low FODMAP diet reduces approximately by half to a total of 17.7 g of FODMAP [12].

Low FODMAP diets have raised concerns as they can alter the microbiota towards an undesired composition. These changes include a reduction in health beneficial *Bifidobacteria*[12–14] and Clostridial species producing SCFAs as well as an increase in bacteria such as *Ruminococcus torques*, previously associated with IBS[15]. This patient group has been shown to have a dysbiotic microbiota[16] and further compromising the composition raises concern.

Testing for the complex interactions between microbiota and intestinal physiology has been limited by the lack of appropriate and non-invasive techniques. MRI of the gut allows non-invasive assessment of luminal content. We have previously demonstrated that it provides a simple and reproducible method for measuring colonic volume and whole gut transit[17, 18]. GI transit and microbiota composition were found to be interrelated in a humanized mouse model, with diet independently affecting both[19]. While conversion of FODMAPs to colonic gas by fermentative bacteria may be an important mediator of symptom development, it is unclear whether breath gas excretion is a good marker of colonic gas production and distension[20]. In a controlled, crossover feeding study with both IBS and healthy subjects, it was demonstrated that a high FODMAP diet increased breath hydrogen production compared to a diet low in FODMAPs in both healthy and IBS patients[21].

Thus far there are no studies of the effect of low FODMAPs on colonic volumes, metabolites and microbiota in healthy subjects. Therefore our aim was to understand the impact of a low FODMAP diet on colonic contents and transit and how this altered the microbiota and their metabolites. We conducted a double-blind clinical trial where healthy subjects consumed a low FODMAP diet and were supplemented with either maltodextrin or oligofructose daily. We selected subjects who were not already excluding FODMAPs from their diet and who did not have unstable microbiota. Therefore, we concentrated on healthy subjects since IBS patients are known to have unstable and dysbiotic microbiota and GI physiology, and often avoid foods with FODMAPs, potentially confounding the effect of a controlled intervention.

## Materials & methods

The study was a single centre, parallel group, double-blind, randomised controlled trial. Ethical approval was granted by the Ethics committee of the School of Medicine, University of Nottingham (A14082014 and Clinical Trial Registry number: NCT02259465).

### Subjects

**Fig 1:**
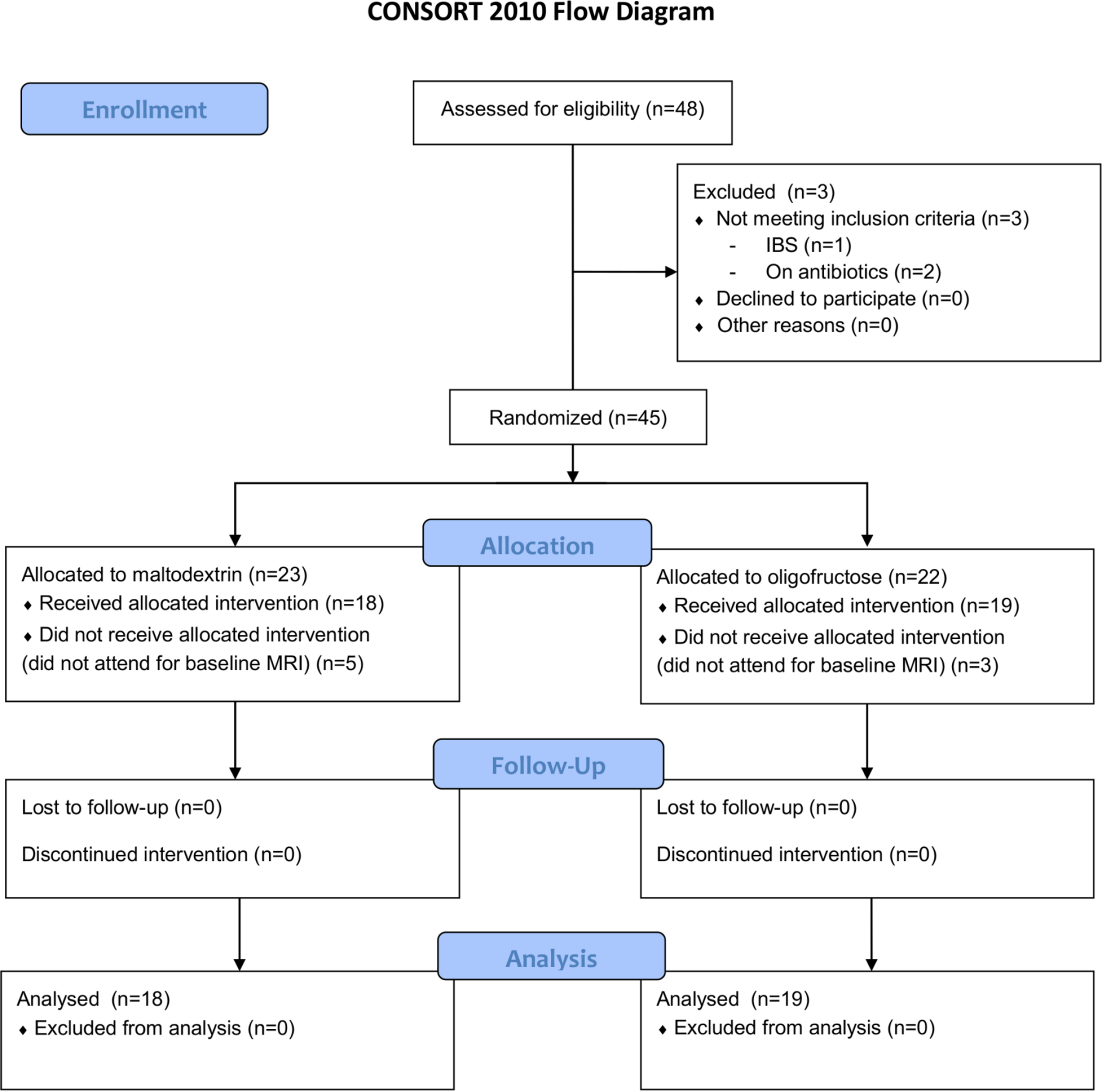
Study CONSORT diagram.

Participants were ≥18 and gave written, informed consent prior to inclusion. Principal exclusion criteria included pregnancy, inability to comply with the diet, any pre-existing gastrointestinal disorder or a positive diagnosis of irritable bowel syndrome based on the Rome III questionnaire, use of antibiotics or probiotics in the preceding 8 weeks, contra-indication to MRI scanning and use of pharmacotherapy likely to alter intestinal motility. The study CONSORT diagram is shown in Fig 1.

## Procedures

**Fig 2:**
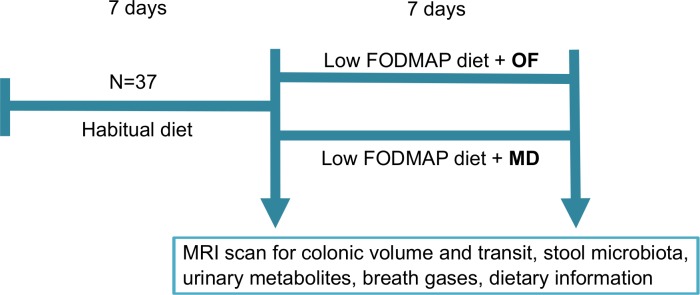
A schematic diagram of subject flow through the study.

A schematic diagram of the study is shown in Fig 2. All participants completed a 7-day food diary. On the morning of day 7 they provided a stool sample and swallowed transit markers for the measurement of whole gut transit time as previously validated[22]. The study dietitian trained subjects in a low FODMAP diet. For the next 24 hours subjects were provided with a standard food package (S1 Table). Subjects also made a 24-hour urine collection, abstained from alcohol and did not eat after 8pm. The next morning, they underwent a fasting MRI scan and gave a breath sample to measure hydrogen and methane content (GastroCH_4_eck™, Bedfont, UK). Subjects then started a 7-day low FODMAP diet. During this time they kept a second food diary. Stool, urine and breath collection and MRI were repeated at the end of the diet. In the last 24 hours of the diet a standard food package specifically low in FODMAPs was provided (S1 Table).

### Randomisation and intervention

All subjects supplemented the low FODMAP diet with 14 g of the added carbohydrate (7 g twice daily). They were randomised to either oligofructose (OraftiP95, Beneo, Germany) or maltodextrin (the Hut Group, Northwich, UK) 1:1 in blocks of 6 using a remote, internet based system. Randomisation was performed by staff members independent of the research team who then dispensed over 100g of supplement of identical colour in opaque plastic containers. Containers were returned at the end of the week to assess adherence based on change in container mass. Subjects, researchers and laboratory staff remained blind to the allocated intervention during analysis.

### Analytical methods

#### Nutrient and FODMAP content

Data from food diaries were analysed with Dietplan 6 P3 (Forestfield Software, Horsham, UK). Energy and nutrient intakes were based on McCance and Widdowson’s Nutrient databank (7^th^ Edition)[23] and FODMAP intakes were calculated from published literature (S1 Methods).

#### Stool microbiota

Faecal samples were stored at -80°C before DNA extraction with a standard previously validated method[24, 25]. Amplicons of the V3-4 region of the 16S rRNA gene were generated by PCR and sequenced with the Illumina MiSeq platform. The mare pipeline was used for sequence pre-processing[26]. Briefly, the forward reads were quality filtered and trimmed to a uniform length (150bp) before clustering and chimera filtering with USEARCH[27]. Taxonomy was assigned to reads with UTAX using the Silva 16S database[28]. The total microbial load[29] and the abundance *of Methanobrevibacter smithii*[30] was determined with qPCR as described previously. The sequencing data are publicly available in the European Nucleotide Archive (ENA, acc.no. PRJEB25589).

#### Metabolomics

Urine metabolites were analysed using liquid chromatography-high resolution mass spectrometry (LC-HRMS). Each sample was assigned ‘aggregate metabolite scores’ for lipids, amino acids and carbohydrates by associating peaks with potential matches in the Human Metabolome Database (HMDB)[31] (S1 Methods).

SCFA analysis of thawed faecal samples was carried out by gas chromatography–mass spectrometry (GC-MS) (S1 Methods).

#### MRI

All scans used a 1.5T Achieva MRI scanner (Philips, Best, The Netherlands). Colonic volume was measured as described previously[17]. Whole gut transit time was evaluated using the weighted average position score (WAPS) as previously validated[22]. Automated colonic gas quantification was also attempted (S1 Methods).

### Statistical analysis

Statistical analysis was performed using R version 3.2.4 (R Foundation for Statistical Computing, Vienna). Results are shown as mean (standard deviation, SD) unless otherwise stated. The primary endpoint of the clinical trial was the effect of the intervention on the percentage change from baseline in colonic volume. A previous 4-period crossover study in a similar population[18] found a coefficient of variation in fasting colonic volume of 17%. The required sample size to detect a difference between groups of 15% was 36 (power 80%, alpha = 0.05 two-sided). A sample size of 45 was planned to allow for a withdrawal rate of 20%.

Microbiota and metabolomics data were exploratory to analyse the impact on composition of the dietary intervention and also provide evidence of adherence. The microbiota profiles were generated without rarefaction and analysed with package mare (Microbiota Analysis in R Easily) [32]. Variation in read counts between samples was controlled for by taking the number of reads as an offset in all statistical models. The significant effect of study groups and the associations between physiological variables (e.g. breath gases and colonic volume) and the microbiota was tested using generalized linear mixed models assuming negative binomial distribution of the data, fitting to each taxon, taking into consideration that the data was zero-inflated. This was calculated using the glmmADMB package of R. In the conducted linear models, the subjects’ age and BMI were accounted for as confounding variables. The obtained p-values were corrected for multiple testing with false discovery rate (FDR, Benjamini–Hochberg [33]) and adjusted p-values (adjust.P) below 0.1 were considered significant.

## Results

The CONSORT flowchart is shown in Fig 1. Altogether 37 subjects completed the study. Baseline characteristics and endpoint data for study participants in both groups are shown in Table 1.

**Table 1:** Participant characteristics and endpoints.

	Maltodextrin			Oligofructose		
Number of subjects	18			19		
Age (years)	23.5 (2.9)			26.5 (12.2)		
Gender (M/F)	14/4			11/8		
Height (m)	1.69 (0.09)			1.73 (0.11)		
Weight (kg)	67.8 (13.6)			67.7 (10.1)		
Body Mass Index (kg/ m^2^)	23.5 (2.9)			22.5 (2.9)		
Supplement compliance (%)	104 (range 91–133)			117 (range 105–132)		
	**BL**	**PI**	**p-value**	**BL**	**PI**	**p-value**
Total colonic Volume (ml)	650 (179)	740 (242)	**0.04**	693 (151)	802 (146)	**0.01**
ascending (ml)	219 (53)	233 (67)	0.34	238 (66)	273 (72)	**0.01**
transverse (ml)	259 (105)	307 (140)	0.07	273 (83)	317 (86)	**0.03**
distal (ml)	171 (73)	201 (87)	0.14	180 (59)	206 (52)	**0.05**
sigmoid	0 (0)	0 (0)	-	3 (11)	8 (33)	0.33
Transit Score (a.u.)	2.09 (1.40)	2.05 (1.44)	0.82	1.51 (0.73)	1.33 (1.07)	0.24
Breath Hydrogen (ppm)	16.8 (12.8)	6.3 (6.3)	**0.006**	19.8 (23.4)	36.3 (32.2)	0.08
Breath Methane (ppm)	20.6 (30.5)	20.0 (33.7)	0.86	19.7 (26.1)	14.6 (26.5)	0.24

Demographic and study endpoint data for study participants. There was no statistical difference between the study groups at baseline. BL = baseline, PI = post-intervention. Data shown as mean (SD) with no adjustment made for multiple comparisons.

### Dietary analysis and adherence to the intervention

**Fig 3:**
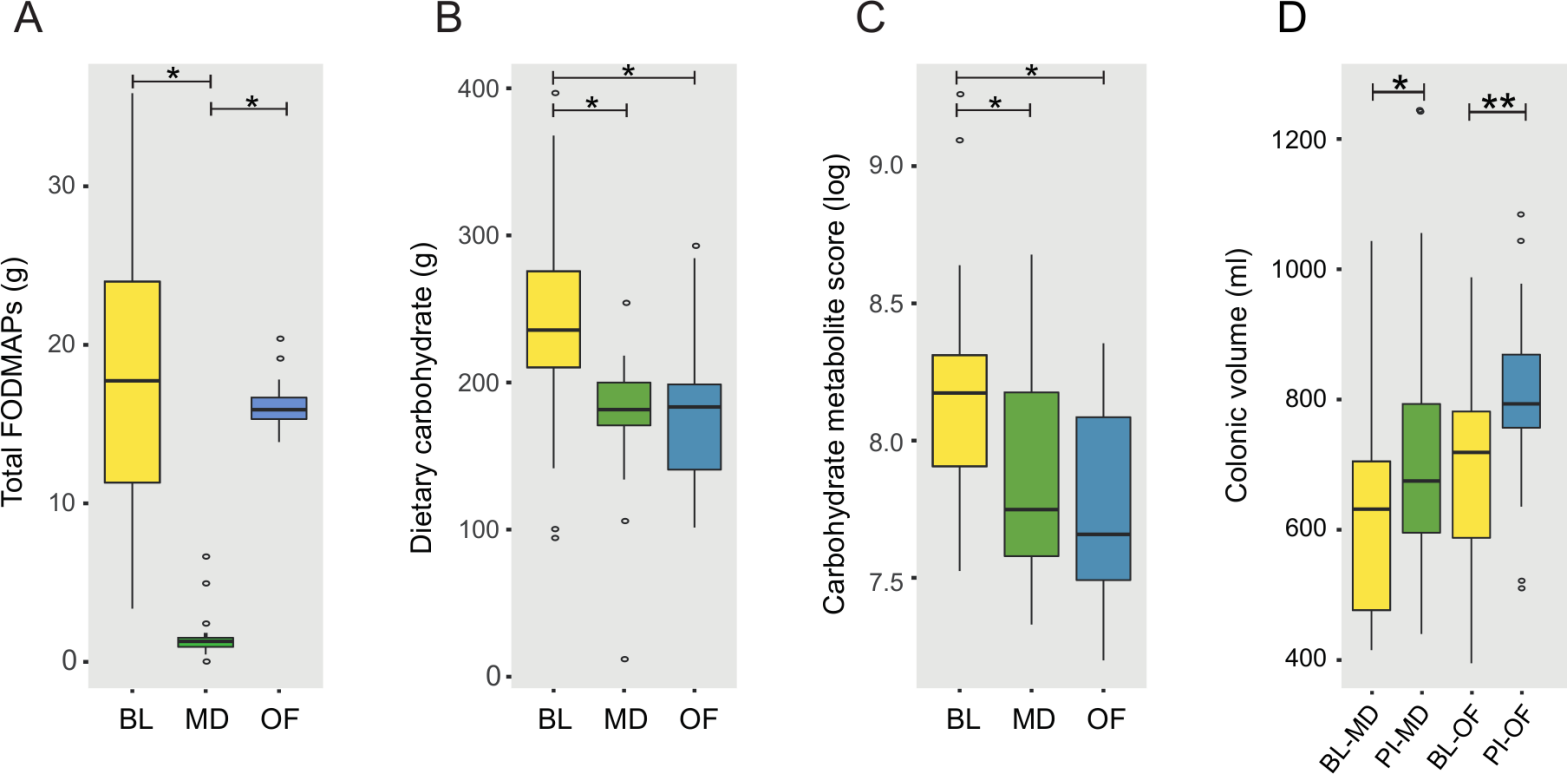
Changes in carbohydrate intake, metabolites and colonic volume. A) Mean daily FODMAP intake (g), including oligofructose supplementation. B) Mean daily carbohydrate intake (g), C) Aggregate metabolite score for ‘Carbohydrates and carbohydrate conjugates’, D) Change in colonic volume post-intervention; BL = baseline, PI = post-intervention, OF = oligofructose group, MD = maltodextrin group. * p < 0.05.

No major deviations from the low FODMAP diet were identified. Daily FODMAP intake decreased from baseline (BL) in the maltodextrin group (mean 17.7g (SD 7.4) to 1.7g (1.6), P<0.01) while remaining constant in the oligofructose group due to the supplement, (18.9g (8.0) to 16.5g (1.6), Fig 3A). Mean supplement compliance was 113 (21.8) % expected supplement usage by weight.

Detailed analysis of the dietary intake revealed changes aside from FODMAP intake during the intervention. For both groups reductions in mean daily carbohydrate (Fig 3B) and total sugar were observed, but no change in fat or protein intake (S2 Table).

#### Urinary metabolites indicate dietary adherence

Urinary metabolic profiles provided additional evidence of adherence with the dietary intervention. The aggregate metabolite score for ‘carbohydrates and carbohydrate conjugates’ was reduced from baseline mean 8.2 (0.4) in both the maltodextrin 7.9 (0.4), P<0.05 and oligofructose-groups 7.8 (0.4), P<0.01, Fig 3C, correlating with total dietary carbohydrate intake (r = 0.38, P = 0.05). The participants’ overall metabolic profile deviated significantly between baseline and post-intervention but there was no significant difference between groups (S1 Fig), suggesting that the supplementation could not cancel the effect of the diet. Aggregate metabolite scores for ‘amino acids, peptides and analogues’ and ‘lipids’ were unchanged from baseline, mirroring unchanged intake of dietary protein and fat.

### Colonic volume and transit

The total colonic volume rose in the oligofructose-group by 19.6% with a mean increase of 110ml (95% CI 30ml to 190ml, P = 0.01). However, volume also increased in the maltodextrin-group, by 15.5% with a mean increase of 90ml (95% CI 6ml to 175ml, P = 0.04, Fig 3D). When separating these into colonic compartments there was a significant increase in the ascending (mean 35ml, 95% CI 9ml to 61ml, P = 0.01), transverse (mean 44ml, 95% CI 4ml to 84ml, P = 0.03) and distal colon (mean 26ml, 95% CI 0ml to 52ml, P = 0.05) only in the OF group (Table 1). There was no significant difference between the groups. Transit scores did not change significantly from baseline in either groups, with no significant difference between them.

### Effects of the intervention on microbiota composition

**Fig 4:**
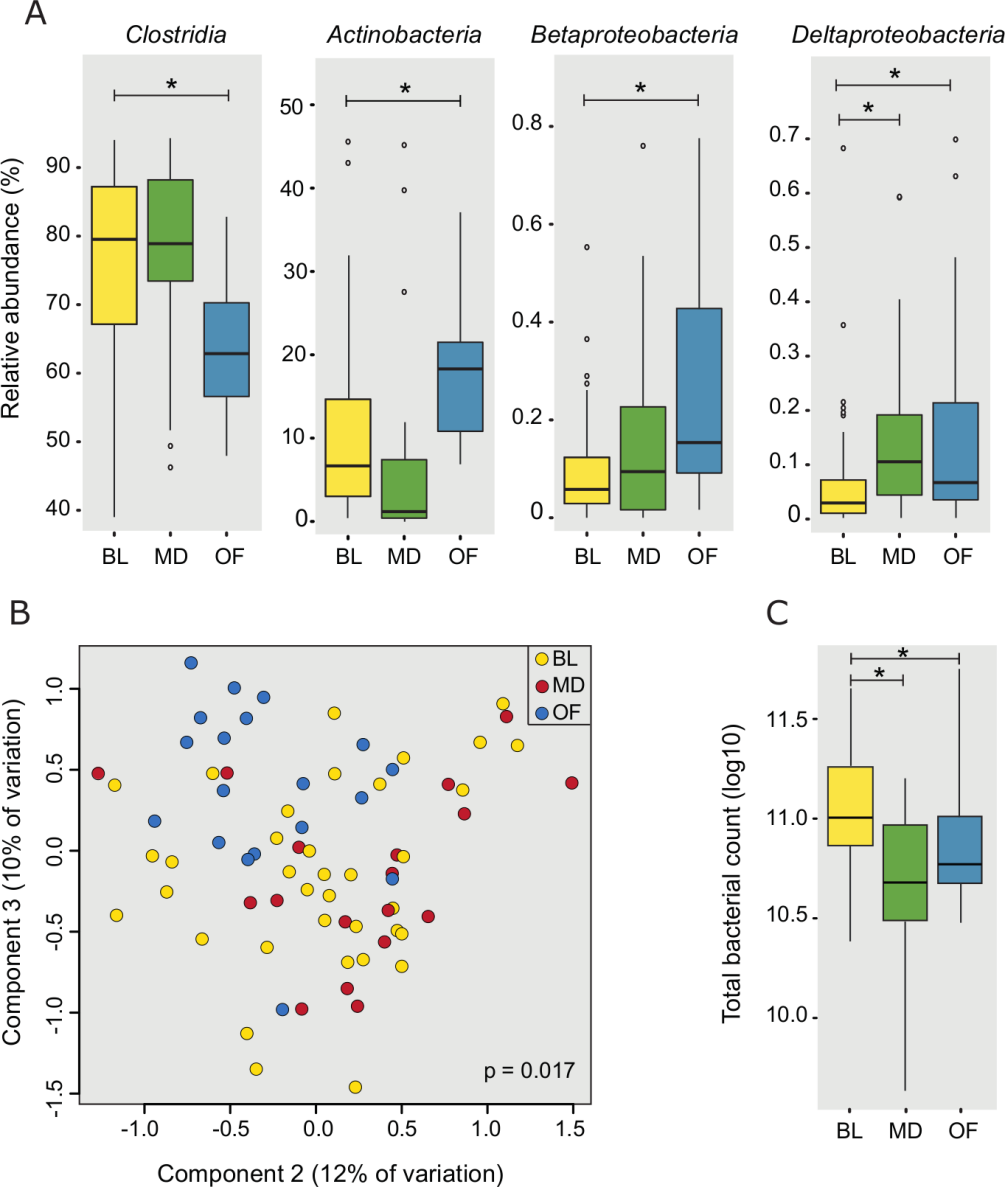
Changes in the microbiota from baseline with the dietary interventions. A) Significant changes at class-level; B) Principal coordinates analysis of sample profiles at genus-level; C) Change in total bacterial count (log^10^/g). BL = baseline, OF = oligofructose group, MD = maltodextrin group. * p < 0.05.

There was a significant drop in the total microbial load after the diet from baseline (11.02 log^10^, SD = 0.27) in both the maltodextrin-group (10.66 log^10^, SD = 0.40, P<0.05) and oligofructose-group (10.87 log^10^, SD = 0.32, P<0.01, Fig 4C).

Principal coordinates analysis indicated that the microbial profiles differed post-intervention between the groups, accounting for 7% of the microbial variation. Even at class level, significant changes in the microbiota were observed in both groups (Fig 4A).

In the maltodextrin-group, a significant reduction in *Actinobacteria* (fold change (fc) = 0.75, FDR corrected adjusted.P<0.01) and increase in *Deltaproteobacteria* (fc = 2.25, adjust.P<0.01) were observed post-intervention. These changes were driven at genus-level by a decrease in *Bifidobacterium* (fc = 0.74, adjust.P = 0.19) and increase in *Bilophila* (fc = 2.34, adjust.P<0.01) respectively. While the overall proportion of *Clostridia* was unchanged, several genera within the family *Ruminococcaceae* were increased from baseline, including *Anaerotruncus* (fc = 1.77, adjust.P<0.05), *Flavonifractor* (fc = 2.21, P<0.01), *Oscillibacter* (fc = 1.85, adjust.P = 0.05) and *Oscillospira* (fc = 3.07, adjust.P<0.01).

In the oligofructose-group, an increased abundance of three classes were observed; *Actinobacteria* (fc = 1.79, adjust.P<0.01), *Betaproteobacteria* (fc = 2.61, adjust.P<0.01) and *Deltaproteobacteria* (fc = 2.25, adjust.P<0.01), while the abundance of *Clostridia* (fc = 0.84, P<0.01) and *Erysipelotrichi* (fc = 0.21, adjust.P<0.05) decreased. The increase in Actinobacteria was due to an increase in *Bifidobacterium* (fc = 1.80, adjust.P = 0.06) while the reduction in *Clostridia* appeared to be predominantly due to a reduction in the family *Lachnospiraceae*, particularly *Anaerostipes* (fc = 0.59, adjust.P = 0.09) and *Blautia* (fc = 0.58, adjust.P<0.01). Within the *Deltaproteobacteria*, *Bilophila* was increased (fc = 2.04, adjust.P = 0.1).

No significant difference was detected in the abundance or prevalence of methanogens post-intervention in either group (BL:5.03 (2.33); maltodextrin:5.24, (2.41); oligofructose:4.66, (2.05), copies 16S/μl of template log^10^). The complete list of genus-level changes following the intervention is shown in S3 Table.

### Products of metabolism and associations with the microbiota

#### Alterations in breath gases

**Fig 5:**
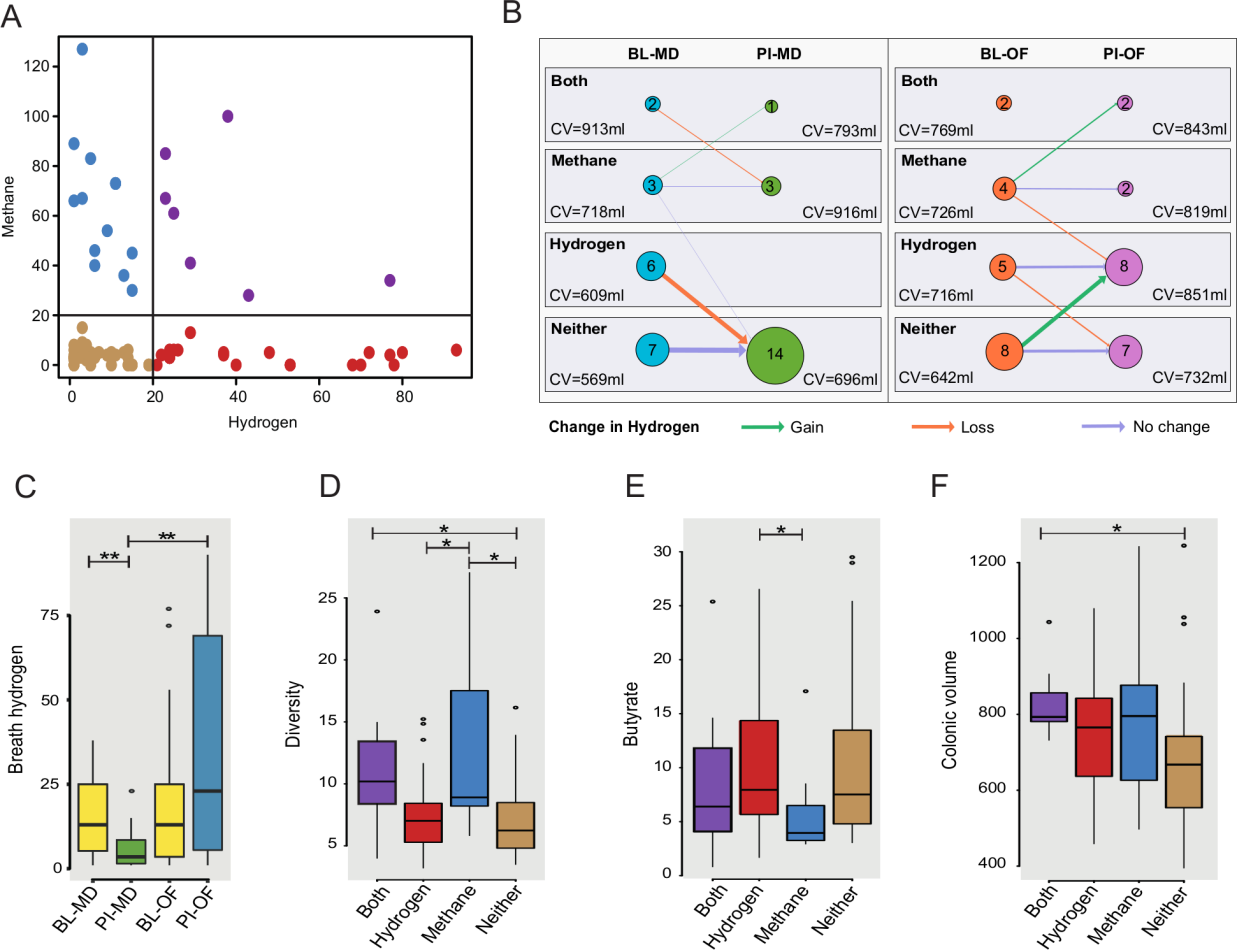
Individual variation in detected breath gases and associations to host parameters. A) Subjects could be categorised into four groups depending on their breath gas: hydrogen & methane >20 ppm (Both), hydrogen & methane < 20 ppm (Neither), only hydrogen > 20 ppm (Hydrogen) or only methane > 20 ppm (Methane), the data shows both time points; B) Transition between gas groups for subjects following the intervention. Node sizes and labels indicate the number of subjects in each group. Arrow weights denote the number of subjects changing groups post-intervention while arrow colours indicate the change in breath hydrogen represented by the transitions. Mean colonic volumes (CV) for each subgroup are shown; C) Divergence in breath hydrogen (ppm) following the intervention; Associations between gas groupings and D) microbial diversity (inverse simpson index), E) butyrate (μmol/g wet stool) and F) colonic volume (ml), data for both time points included. BL = baseline, PI = post-intervention, OF = oligofructose, MD = maltodextrin, H2 = Hydrogen, CH4 = Methane. * p < 0.05, ** p < 0.01.

The groups showed a divergent change in fasting breath hydrogen amounts with an increase in the oligofructose group of 16ppm (95% CI -2 to 35) after the diet and decrease in the maltodextrin group of 11ppm (95% CI 3 to 18). The difference between groups was 27ppm (95% CI 7 to 50, P<0.01, Fig 5C). The methane levels did not change significantly from baseline or differ between groups. We tested if the changes in breath gas levels could be associated with the microbiota composition and found a significant negative association between increased breath hydrogen and the abundance of *Blautia* from the *Lachnospiraceae* family (P = 0.04).

As some individuals appeared to produce methane in preference to hydrogen, subjects could be divided into four groups by the levels of gas detectable in breath at baseline, which allowed more detailed analysis of changes in gas production: hydrogen only, methane only, both or neither (Fig 5A). At baseline the biggest sub-group (15/37) had neither gas in their breath above a clinically relevant threshold of 20ppm while 11 had raised hydrogen, 7 raised methane and 4 had both gases raised. In the maltodextrin-group, all 8 subjects that had recorded fasting breath hydrogen >20ppm at baseline dropped their levels below this threshold (Fig 5B). In the oligofructose group the trend was opposite where 6 subjects with breath hydrogen <20ppm at baseline, had breath hydrogen >20ppm after the diet.

#### Associations between gas groupings and other parameters

Associations were sought between inter-individual variation in breath gas production and the microbiota composition. Samples taken from subjects with lower methane levels had significantly lower microbial diversity compared to those with high levels of breath methane (Fig 5D). Moreover, 10 genus-level taxa were associated with breath gas groupings (S2 Fig), including increased abundance of *Akkermansia*, *Ruminococcus* and *Methanobrevibacter smithii* in association with increased breath methane, and increased abundance of *Veillonella* with increased breath hydrogen.

In addition to microbial associations, subjects with hydrogen levels >20ppm were observed to have significantly higher concentrations (p = 0.05) of butyrate in their stool at that time point (Fig 5E) than those with lower levels. Colonic volumes were also significantly larger in subjects with above-threshold levels of both hydrogen and methane compared to those where neither gas reached threshold (P<0.05, Fig 5B & 5F).

#### Short chain fatty acid concentrations and the microbiota

There was no significant change in the levels of SFCA post-intervention or between groups (S4 Table). Therefore, we sought to find associations between the microbiota composition and stool SCFAs. In total 21 genus-level bacterial taxa were found to be significantly associated. Of note, in the family *Lachnospiraceae*, *Roseburia* was positively associated with increased butyrate (P<0.01) and acetate (P<0.05), while a member of the same family, *Anaerostipes* showed negative associations with isobutyrate (P = 0.09) and valeric acid (P<0.05) (S5 Table).

### Correlations between colonic volume and other physiological parameters

**Fig 6:**
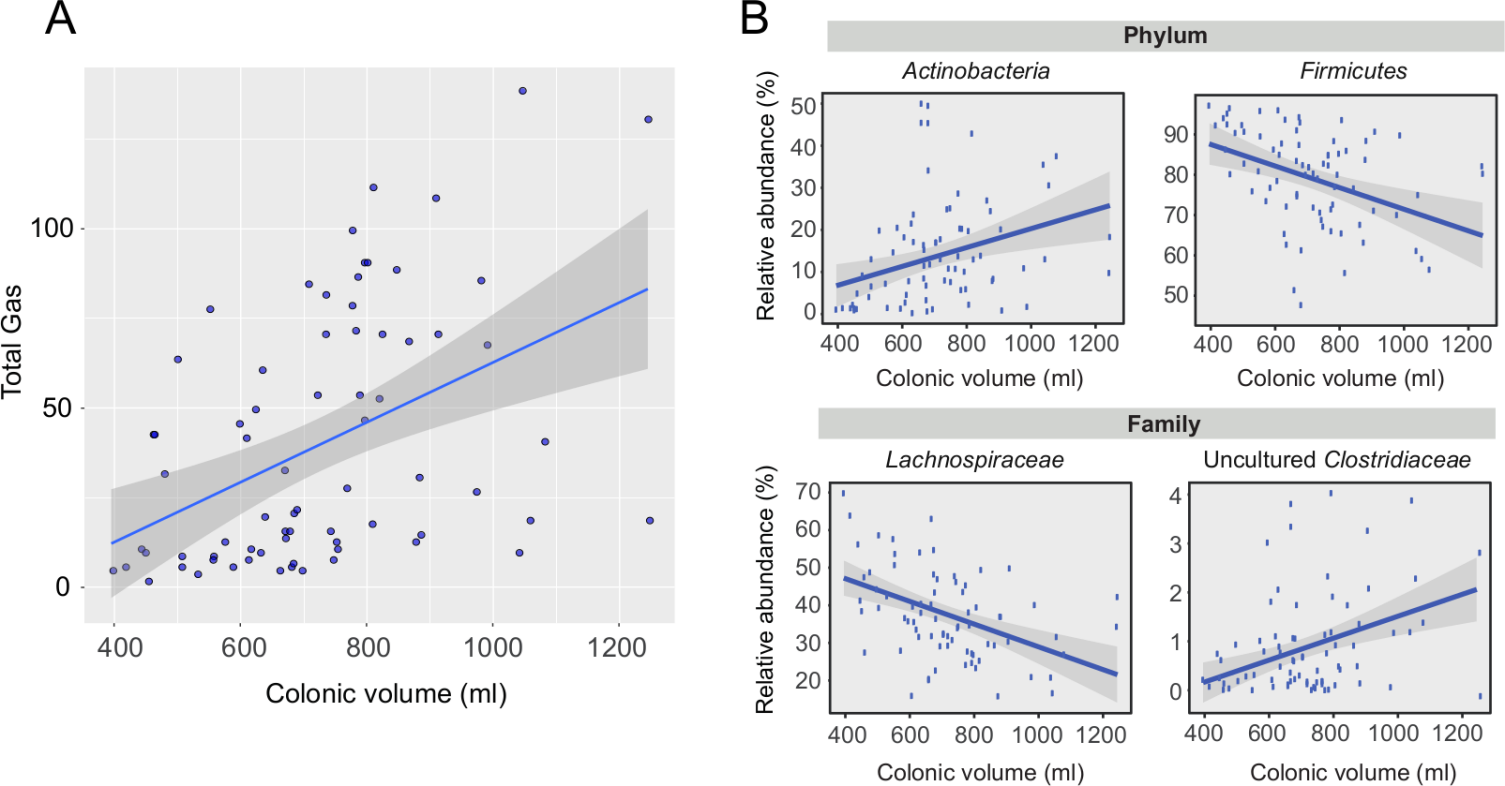
Colonic volume and associations with breath gas and microbiota. A) Correlation between colonic volume and total breath gases (hydrogen and methane, ppm) at both time points (rho = 0.51, p < 1 x 10^−5^); Associations between colonic volume and the microbiota on B) phylum-level and C) family-level over both time points. BL = baseline, PI = post-intervention, OF = oligofructose, MD = maltodextrin.

Colonic volume did not correlate with breath hydrogen (baseline r = 0.16, P = 0.34, post-intervention r = 0.26, P = 0.12) and only weakly with breath methane (baseline r = 0.32, P = 0.06, post-intervention r = 0.32, P = 0.06). However, the two breath gases combined correlated strongly with colonic volume both at baseline (r = 0.43, P<0.01) and post-intervention (r = 0.58, P<0.001) suggesting a potential biological association between total gas production and colonic volume (Fig 6A). Colonic volume also correlated significantly with transit score both at baseline (r = 0.41, P = 0.01) and after intervention (r = 0.37, P<0.05, S3 Fig).

#### Associations between colonic volume and the microbiota

Given the potential role of the microbiota in influencing colonic volume both directly, through gas production, and indirectly through other mechanisms, we looked for associations between the microbiota and colonic volume. Interestingly, colonic volume correlated significantly with overall microbial diversity at baseline (r = 0.62, adjust.P<0.001) but not post-intervention (r = 0.27, adjust.P = 0.11, S3 Fig).

We constructed generalised linear models to explore associations between bacterial taxa and colonic volume, accounting for diet. Beginning at phylum-level, we found that the *Actinobacteria* were positively associated with volume (adjust.P<0.05) while *Firmicutes* were negatively associated (P<0.05, Fig 6B). To determine the organisms driving these associations we constructed the same models for both family and genus-level taxa. At family-level, two taxa from the *Firmicutes* phylum had significant associations: *Lachnospiraceae* (adjust.P<0.01) were negatively associated with volume while uncultured *Clostridiaceae* (adjust.P<0.05) were positively associated (Fig 6C). Furthermore, at genus-level the uncultured *Clostridium* were again positively associated (adjust.P<0.05) and *Roseburia* from the *Lachnospiraceae* family were negatively associated (adjust.P<0.05, S3 Fig).

## Discussion

The current study is the first to assess colonic contents using MRI, concurrently with abundance of colonic metabolites and a comprehensive characterisation of the intestinal microbiota, in order to understand the effect of dietary changes. We showed that a low FODMAP diet in healthy subjects had an impact on intestinal microbiota composition, levels of breath hydrogen and colonic volume.

Adherence to the exclusion diet was confirmed by a number of methods including food diaries, supplement use and urinary metabolites. We showed a significant reduction in FODMAP intake in both groups before supplementation, but also revealed a fall in total carbohydrate intake accompanied by a reduction in urinary metabolites associated with carbohydrate metabolism. This could be explained by the avoidance of high FODMAP containing snacks. While low FODMAP diets aim to replace fermentable non-starch polysaccharides with alternatives, this will inevitably lead to some structural changes in overall intake.

The changes in the intestinal microbiota introduced by low FODMAP diets has recently raised concern, especially the reduction in health-beneficial bifidobacteria[13, 34]. Previously this was studied only in IBS patients, however we have now replicated this finding in healthy volunteers, showing that restricting FODMAP intake reduced the relative abundance of *Actinobacteria*, predominantly *Bifidobacteria*, in the maltodextrin-group but increased abundance in the oligofructose-group due to the bifidogenic effect of oligofructose[35, 36]. We also showed total microbial amount was reduced, reaching significance in the maltodextrin-group, replicating previous research in IBS patients[13].

The other taxa affected by the dietary intervention included a significant reduction in *Clostridia* in the oligofructose-group, particularly *Lachnospiraceae*, many of which are butyrate producers. This raises the possibility that these taxa may have been displaced by oligofructose-induced proliferation of *Bifidobacteria*. McIntosh et al. reported reductions in *Lachnospiraceae* when IBS patients followed a low FODMAP diet[34]. In the maltodextrin-group, an increase in *Ruminococcaceae* appeared to accompany the reduction in *Bifidobacteria*. Such changes may reflect the effect on the microbial ecosystem as a whole of attempts to alter the abundance of a single target group. While diverging effects were observed in microbial composition of the study groups, a twofold increase in *Deltaproteobacteria*, particularly *Bilophila*, abundance was observed in both, possibly indicating an adverse effect of the change in diet. Sulphate reducing bacteria (SRB), such as *Bilophila* and *Desulfovibrio*, are potential pathogens, competing with other lactate-utilising bacteria such as *Anaerostipes* in order to produce hydrogen sulphide which can be toxic to colonocytes[37]. Although the increase in protein intake on the low FODMAP diet was small, bile tolerant bacteria such as *Bilophila* have been associated with a meat based diet so could have been stimulated by substituting carbohydrates with animal protein[38].

Our breath hydrogen results are consistent with previous studies showing a rise in the abundance of hydrogen when subjects were supplemented with oligofructose[39] and a fall on a low FODMAP diet[21]. Interestingly, it has been shown that *Lachnospiraceae*, notably *Blautia*, are capable of autotrophic conversion of hydrogen and carbon dioxide to acetate, and this may partly explain the increased breath hydrogen seen in the oligofructose-group associated with reduced *Blautia* abundance.

As there is a recognised heterogeneity in response to low FODMAP diets in IBS patients[40], we sought to better understand this phenomenon by investigating the inter-individual response to the intervention in relation to breath gases. We noticed that some subjects with detectable amounts of breath gases appeared to express either hydrogen or methane gas in their breath, creating four distinct subgroups allowing us to separate subjects according to physiological response to the diet. We recognised that the majority of the changes introduced by the diet were in relation to hydrogen, with all of the baseline hydrogen producers in the maltodextrin group losing this production post-intervention, whereas in the oligofructose group, the majority, but not all, either retained or gained hydrogen production.

Although methane production did not change in either group, there was a subset of subjects with high methane production and distinct characteristics, including higher microbial diversity, higher colonic volume, reduced stool butyrate and increased abundance of methane-producing *Methanobrevibacter smithii*. Surprisingly, methane production was strongly associated with higher microbial diversity. We hypothesise that the presence of methanogens might facilitate distinct niches for elements of the microbiota that would otherwise not exist and could reflect a diversion of microbial fermentation end-products away from the production of hydrogen and butyrate, towards methane.

**Fig 7:**
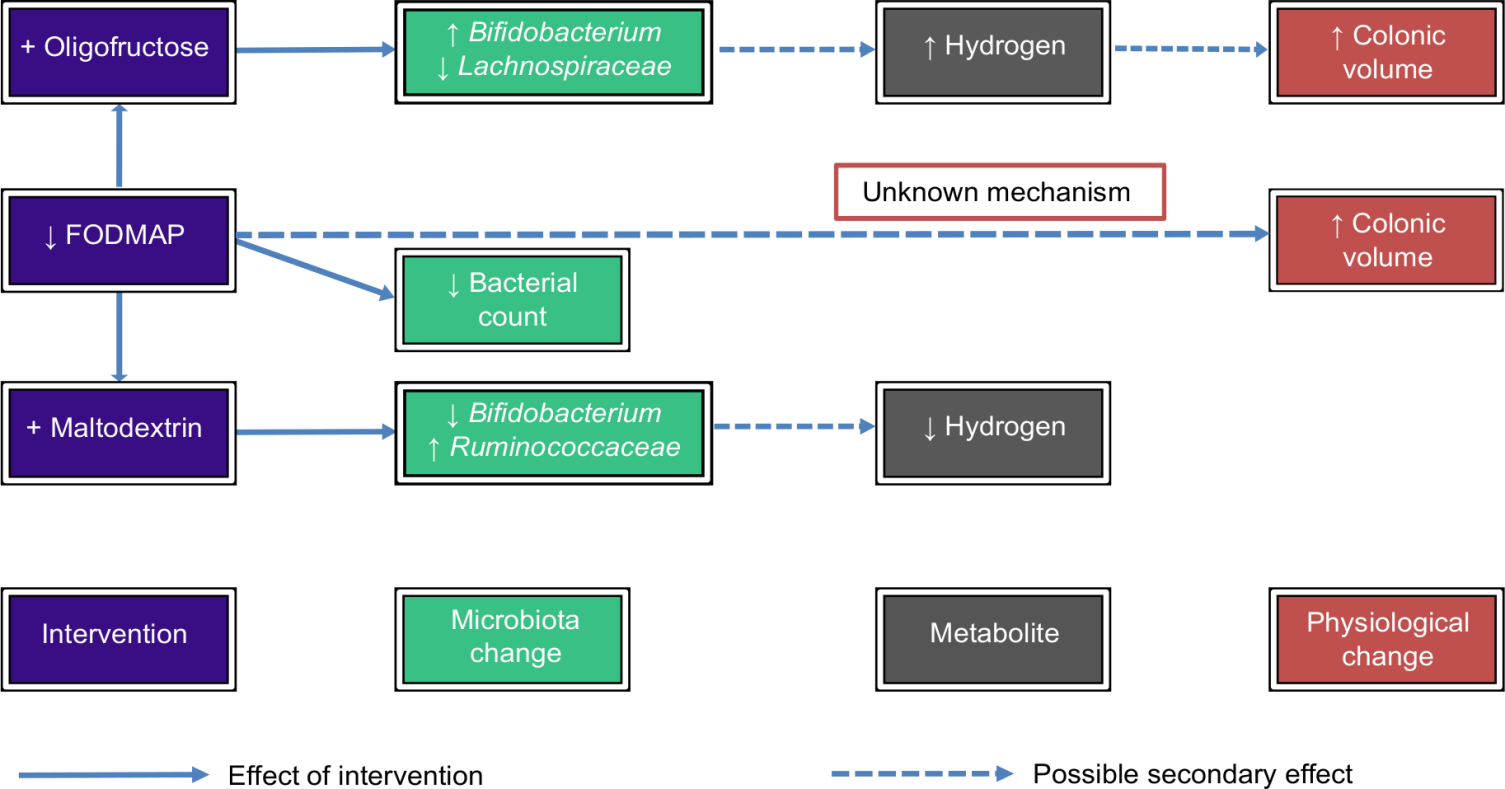
Summary of potential mechanisms underlying observed changes in the microbiota and colonic volume following the dietary interventions.

Both hydrogen and methane were shown to be related to higher colonic volumes, possibly explaining some of the increase in volume in the oligofructose-group. However, there was also a trend towards increasing volume across all subjects post-intervention, regardless of changes in gas production, which is unexplained. Previously it was thought that a low FODMAP diet would reduce microbial fermentation, gas production and therefore colonic volume thereby relieving pain and bloating in IBS sufferers. We have not found any evidence to support this hypothesis in healthy subjects, raising the prospect that the success of the diet in IBS patients is due to an alternative mechanism, perhaps mediated by the effect of bacterial metabolites on the enteric nervous system. There is clearly a complex interaction between the diet, microbiota changes, colonic metabolites and GI physiology (Fig 7).

In order to better understand the underlying mechanisms for increased colonic volume, we looked for associations between the microbiota and colonic volume. Colonic volume was found to be positively associated with *Actinobacteria* and negatively associated with *Lachnospiraceae*, mirroring the changes in these taxa seen in subjects after taking oligofructose. Specifically, increased *Roseburia* was associated with reduced colonic volume and increased butyrate and acetate. Butyrate may modulate colonic volume by stimulating increased colonic muscle tone or contractility, suggesting another potential effect of the microbiota on colonic volume[41]. While not significant, the slowing of transit could also have contributed to increased volume.

When analysing the microbiota changes and association with other variables, we employed a statistical model accounting for confounding factors and with strict adjustment for multiple comparisons. We also sought to interrogate any positive findings in light of the current literature and potential biological significance. Nonetheless, it should be emphasised that these analyses were exploratory and should be interpreted cautiously, particularly in view of the limited sample size.

Our study complements previous work and emphasises the need to consider diet, colonic environment and intestinal microbiology together in order to interpret their interactions. It also suggests that low FODMAP diets alleviate GI symptoms through reduced microbial fermentation rather than reducing colonic distension. Further work should focus on how the alteration in different groups of colonic microbiota induced by a low FODMAP diet changes specific metabolites which alter colonic function.

## Supporting information

S1 CONSORT checklist Study CONSORT checklist. (PDF)

S1 Fig Variation in urinary metabolite profiles between groups. Principal Coordinates Analysis of urinary metabolite profiles showing separation between baseline and post-intervention samples. BL = baseline, MD = maltodextrin, OF = oligofructose.
(TIF)

S2 Fig Variation in microbial taxa with gas groupings. Data is either shown as relative abundance (% total) for bacteria or amount of the Methanobrevibacter specific nifH-gene per μg template DNA. Gas groupings were defined according to cut-off values; >20 ppm hydrogen and <20 ppm methane (Hydrogen), >20 ppm methane and <20 ppm hydrogen (Methane), >20 ppm hydrogen and >20 ppm methane (Both) or <20 ppm hydrogen and <20 ppm methane (No), data shown for both time points.
(TIF)

S3 Fig Associations between colonic volume and other variables. Data for both time points is shown with linear model fits as lines and shaded areas representing 95% confidence intervals. A) Colonic volume correlates with transit (WAPS) at baseline (r = 0.43, p<0.01), post-intervention (r = 0.37, p<0.05) and overall (r = 0.43, p<0.01). B) Colonic volume correlates with microbial diversity (Inverse Simpson Index) at baseline (r = 0.62, p<0.001) and overall (r = 0.43, p<0.001) but not post-intervention (r = 0.27, p = 0.11). Colonic volume was a significant predictor of microbial abundance for two genera based on generalised linear modelling; C) Uncultured Clostridium (p = 0.08, FDR corrected) and D) Roseburia (p = 0.03, FDR corrected).
(TIF)

S1 Table Details of food package contents. Food package contents provided to study subjects 24 hours prior to MRI scan for baseline (standard food package) and post-intervention (low FODMAP food package).
(PDF)

S2 Table Detailed dietary breakdown. Table of mean daily intake for individual dietary components. The intervention supplements are not included. MD, maltodextrin; OF, oligofructose; BL, baseline; PI, post-intervention. SD values given in brackets. Values which differ significantly from baseline are shown in bold.
(PDF)

S3 Table Detailed microbiota changes. Complete list of significant genus level changes post-intervention compared to the baseline. FDR corrected p-values >0.05 are show in bold.
(PDF)

S4 Table Faecal short chain fatty acid concentrations. Short chain fatty acid concentrations per study group. MD, maltodextrin; OF, oligofructose; BL, baseline; PI, post-intervention. No significant differences between the groups. Values shown are mean (SD) μmol/g wet stool per group.
(PDF)

S5 Table Associations between the microbiota and short chain fatty acids. Statistically significant associations between genus level taxa and short chain fatty acids, across both time points, showing positive or negative associations calculated with linear mixed models.
(PDF)

S1 Methods Supplementary methods. Detailed description of methods for dietary and metabolomics analysis.
(PDF)

S1 Protocol Detailed study protocol. (PDF)

S1 Script R script for microbiota testing. R script for comparing the microbiota to categorical and continuous variables using the package Microbiota Analysis in R Easily (mare). Viewable with any text editor.
(R) Click here for additional data file.

S1 Output Full output of microbiota comparison by study group. Tabulated output from the GroupTest function within mare indicating relative abundance, fold change, raw and corrected p-values for all identified genera by study group.
(XLSX)
