# Supplementary figures and images for: A low FODMAP diet is associated with changes in the microbiota and reduction in breath hydrogen but not colonic volume in healthy subjects
Source: PLoS One. 2018 Jul 26;13(7):e0201410. doi: 10.1371/journal.pone.0201410 (PMC6062106; doi:10.1371/journal.pone.0201410)

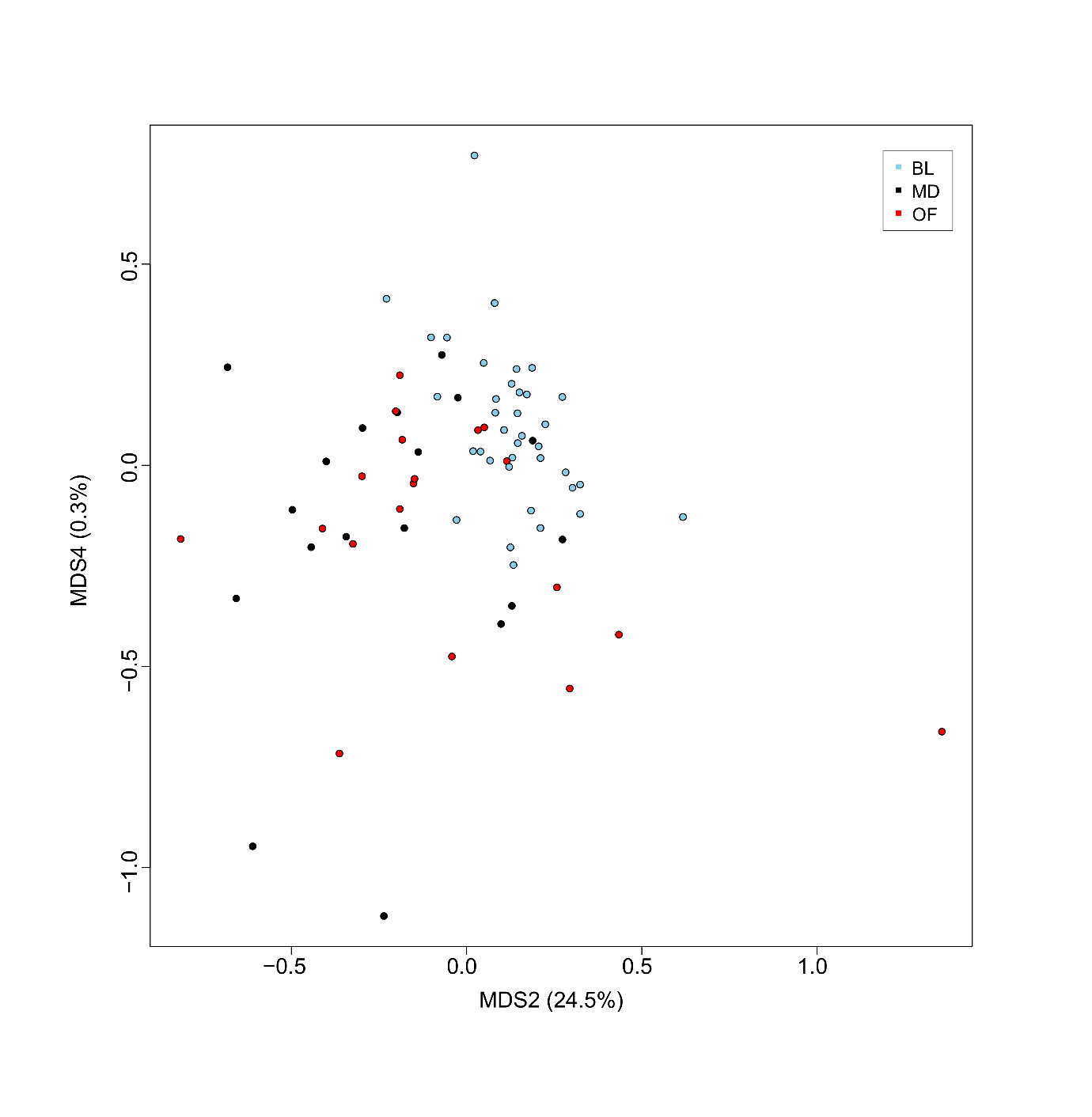

Supplement: S1 Fig — Principal Coordinates Analysis of urinary metabolite profiles showing separation between baseline and post-intervention samples. BL = baseline, MD = maltodextrin, OF = oligofructose. (TIF) [file pone.0201410.s002.tif]

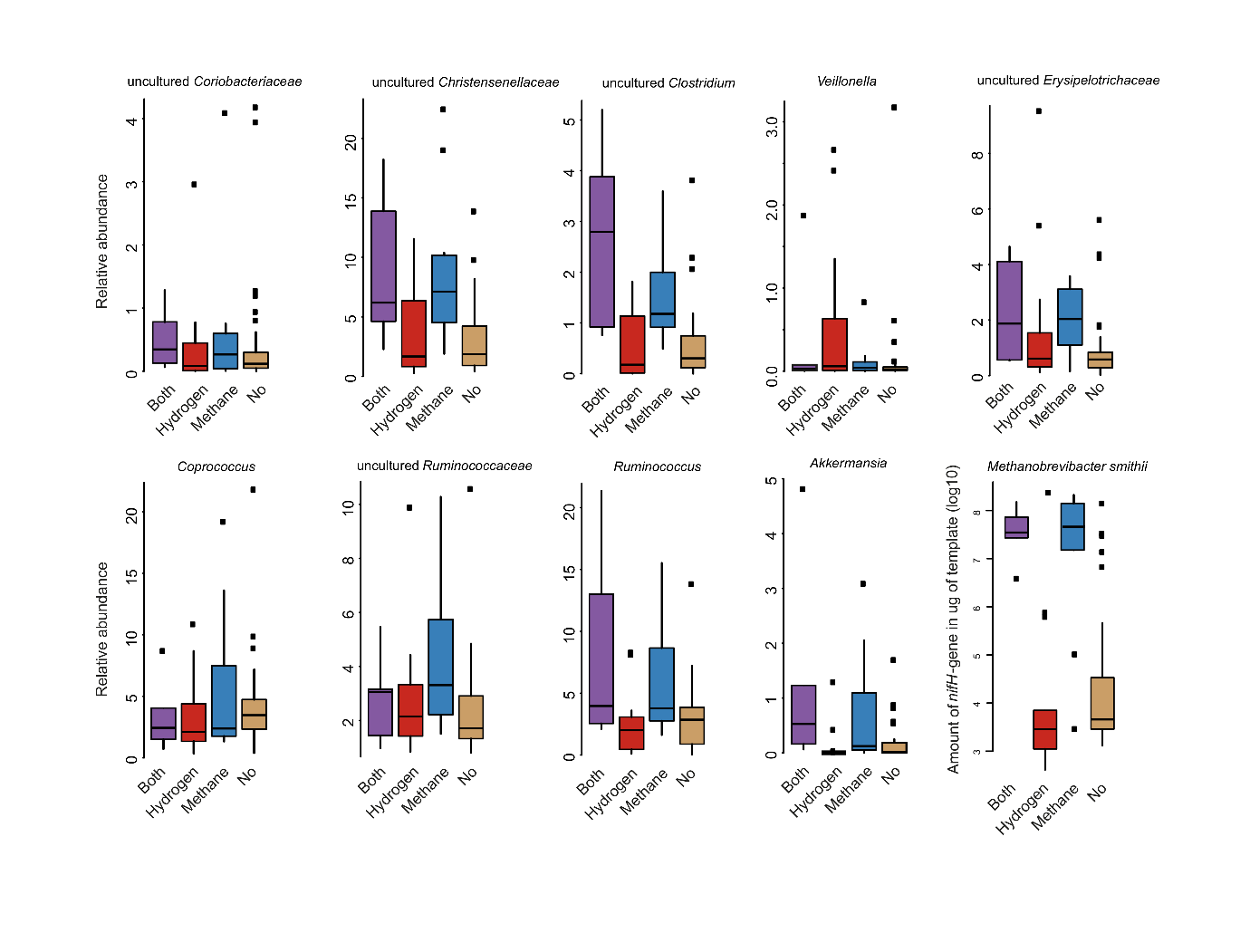

Supplement: S2 Fig — Data is either shown as relative abundance (% total) for bacteria or amount of the Methanobrevibacter specific nifH-gene per μg template DNA. Gas groupings were defined according to cut-off values; >20 ppm hydrogen and <20 ppm methane (Hydrogen), >20 ppm methane and <20 ppm hydrogen (Methane), >20 ppm hydrogen and >20 ppm methane (Both) or <20 ppm hydrogen and <20 ppm methane (No), data shown for both time points. (TIF) [file pone.0201410.s003.tif]

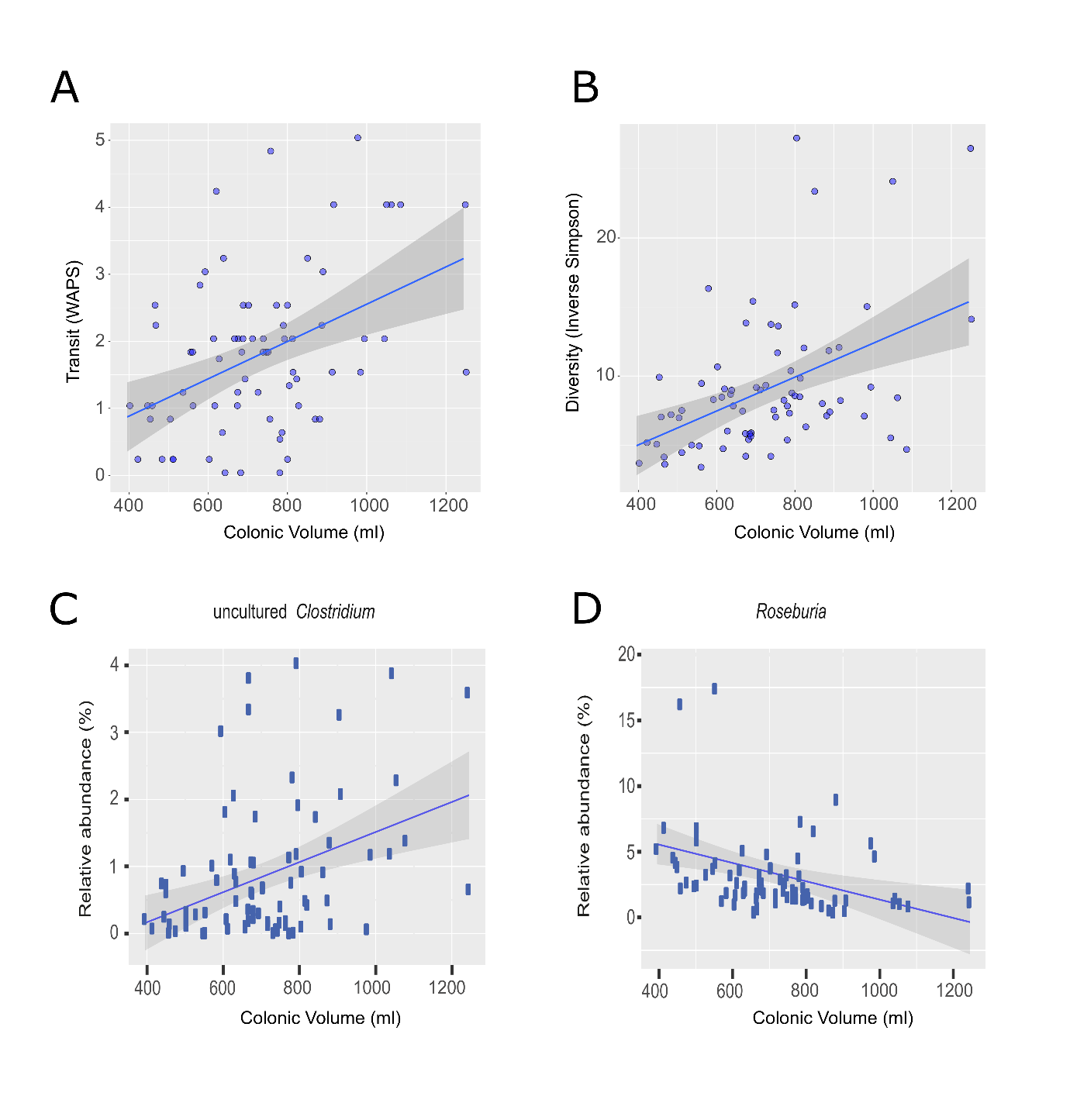

Supplement: S3 Fig — Data for both time points is shown with linear model fits as lines and shaded areas representing 95% confidence intervals. A) Colonic volume correlates with transit (WAPS) at baseline (r = 0.43, p<0.01), post-intervention (r = 0.37, p<0.05) and overall (r = 0.43, p<0.01). B) Colonic volume correlates with microbial diversity (Inverse Simpson Index) at baseline (r = 0.62, p<0.001) and overall (r = 0.43, p<0.001) but not post-intervention (r = 0.27, p = 0.11). Colonic volume was a significant predictor of microbial abundance for two genera based on generalised linear modelling; C) Uncultured Clostridium (p = 0.08, FDR corrected) and D) Roseburia (p = 0.03, FDR corrected). (TIF) [file pone.0201410.s004.tif]
