# Supplementary material for: A low FODMAP diet is associated with changes in the microbiota and reduction in breath hydrogen but not colonic volume in healthy subjects
Source: PLoS One. 2018 Jul 26;13(7):e0201410. doi: 10.1371/journal.pone.0201410 (PMC6062106; doi:10.1371/journal.pone.0201410)
Supplement: S1 Table — Food package contents provided to study subjects 24 hours prior to MRI scan for baseline (standard food package) and post-intervention (low FODMAP food package). (PDF) [file pone.0201410.s005.pdf]

S1 Table. Details of food package contents.

	Breakfast	Lunch	Dinner
**Standard food package**	2 slices medium white bread (Tesco) 72g<br><br>Coffee/ tea 125mL with milk (dash)<br><br>1 portion of jam 20g<br><br>1 portion of butter 5g	Cheese sandwich on white bread 110g<br><br>1 apple<br><br>Ready salted crisps (Walkers) 25g<br><br>Strawberry yoghurt 85g	2 slices bread 72g<br><br>Soup (Heinz) 400g<br><br>Orange juice 85mL<br><br>Gluten-free cake slice 100g
**Low FODMAP food package**	1 sachet Oats So Simple – golden syrup (Quaker) with ½ cup lactose free milk<br><br>Coffee/ tea 125mL with lactose free milk (dash)	2 slices gluten-free bread (Tesco)<br><br>Tuna steak in spring water (John West 'no drain') 120g<br><br>1 sachet mayonnaise (Heinz)<br><br>6 gluten-free oatcakes (Nairn)<br><br>1 medium size banana<br><br>Ready salted crisps (Walkers) 25g	Lean corned beef (Princes) 200g<br><br>Tinned whole carrots in water (Tesco) 300g<br><br>Long grain microwave rice (Tesco) 250g<br><br>2 clementines

Food package contents provided to study subjects 24 hours prior to MRI scan for baseline (standard food package) and post-intervention (low FODMAP food package).
