# Supplementary material for: A low FODMAP diet is associated with changes in the microbiota and reduction in breath hydrogen but not colonic volume in healthy subjects
Source: PLoS One. 2018 Jul 26;13(7):e0201410. doi: 10.1371/journal.pone.0201410 (PMC6062106; doi:10.1371/journal.pone.0201410)
Supplement: S2 Table — Table of mean daily intake for individual dietary components. The intervention supplements are not included. MD, maltodextrin; OF, oligofructose; BL, baseline; PI, post-intervention. SD values given in brackets. Values which differ significantly from baseline are shown in bold. (PDF) [file pone.0201410.s006.pdf]

S2 Table. Detailed dietary breakdown.

Food component	MD-BL	MD-PI	OF-BL	OF-PI
Energy (kcal/d)	2044(474)	1904(648)	2224(539)	2068(744)
Protein (g/d)	81.2(23.7)	91(41.7)	95.1(26)	99.3(46.8)
Fat (g/d)	83.4(24.6)	82(29.7)	90.6(29)	85.3(32)
Carbohydrate (g/d)	234(46.8)	**189(63.5)**	246(72.5)	**200(85.8)**
Sugar (g/d)	102(36.5)	**76(31.6)**	92(31)	**63(31)**
Starch (g/d)	123.7(38.4)	126(39.7)	150(56)	140(55)
Fibre (g/d)	18.9(5.5)	19.6(7.6)	21(7)	19.7(5.8)
Total FODMAPs (g/d)	17.7(7.4)	**1.7(1.6)**	18.9(8.0)	**2.5(1.6)**
Fructans (g/d)	2.4(0.9)	**0.3(0.5)**	2.9(1.4)	**0.2(0.2)**
GOS (g/d)	0.6(0.3)	**0.1(0.3)**	0.8(0.5)	**0.2 (0.3)**
Lactose (g/d)	9.9(6.8)	**0.69(0.8)**	10.6(6.0)	**1.4(1.5)**
Total fructose (g/d)	17.1(8.6)	**11.1(7.0)**	14.6(8.1)	**10.6(7.8)**
Excess fructose (g/d)	3.8(2.4)	**0.4(0.3)**	3.5(2.3)	**0.5(0.3)**
Sorbitol (g/d)	0.7(0.7)	**0.4(0.6)**	0.7(0.5)	**0.9(0.9)**
Mannitol (g/d)	0.2(0.2)	0.7(0.1)	0.3(0.4)	**0.9(0.2)**

Table of mean daily intake for individual dietary components. The intervention supplements are not included. MD, maltodextrin; OF, oligofructose; BL, baseline; PI, post-intervention. SD values given in brackets. Values which differ significantly from baseline are shown in bold.
