# Supplementary material for: A low FODMAP diet is associated with changes in the microbiota and reduction in breath hydrogen but not colonic volume in healthy subjects
Source: PLoS One. 2018 Jul 26;13(7):e0201410. doi: 10.1371/journal.pone.0201410 (PMC6062106; doi:10.1371/journal.pone.0201410)
Supplement: S3 Table — Complete list of significant genus level changes post-intervention compared to the baseline. FDR corrected p-values >0.05 are show in bold. (PDF) [file pone.0201410.s007.pdf]

S3 Table. Detailed microbiota changes.

Genus level taxon	**BL**	**MD**	**OF**	Fold change to BL **MD**	Fold change to BL **OF**
Actinobacteria; Coriobacteriia; Coriobacteriales; Coriobacteriaceae; Eggerthella	0.05 %	**0.15 %**	0.02 %	2.93	0.45
Bacteroidetes; Bacteroidia; Bacteroidales; Porphyromonadaceae; Butyricimonas	0.02 %	0.03 %	**0.05 %**	1.75	2.56
Bacteroidetes; Bacteroidia; Bacteroidales; Porphyromonadaceae; Parabacteroides	0.13 %	**0.37 %**	**0.26 %**	2.91	2.06
Bacteroidetes; Bacteroidia; Bacteroidales; Prevotellaceae; Paraprevotella	0.01 %	0.01 %	**0.07 %**	0.95	5.65
Bacteroidetes; Bacteroidia; Bacteroidales; Prevotellaceae; Prevotella	0.63 %	**0.19 %**	1.57 %	0.30	2.49
Firmicutes; Bacilli; Lactobacillales; Lactobacillaceae; Lactobacillus	0.03 %	0.04 %	**0.14 %**	1.16	4.49
Firmicutes; Clostridia; Clostridiales; FamilyXIIncertaeSedis; Parvimonas	0.00 %	**0.01 %**	0.00 %	23.39	0.74
Firmicutes; Clostridia; Clostridiales; Lachnospiraceae; Anaerosporobacter	0.01 %	**0.00 %**	0.01 %	0.25	0.96
Firmicutes; Clostridia; Clostridiales; Lachnospiraceae; Blautia	7.07 %	6.09 %	**4.12 %**	0.86	0.58
Firmicutes; Clostridia; Clostridiales; Peptostreptococcaceae; IncertaeSedis	0.37 %	0.43 %	**0.21 %**	1.19	0.57
Firmicutes; Clostridia; Clostridiales; Ruminococcaceae; Anaerotruncus	0.23 %	**0.41 %**	0.25 %	1.77	1.09
Firmicutes; Clostridia; Clostridiales; Ruminococcaceae; Flavonifractor	0.40 %	**0.88 %**	0.34 %	2.21	0.85
Firmicutes; Clostridia; Clostridiales; Ruminococcaceae; Oscillibacter	0.08 %	**0.14 %**	0.10 %	1.85	1.34
Firmicutes; Clostridia; Clostridiales; Ruminococcaceae; Ruminococcus	4.38 %	3.40 %	**2.03 %**	0.78	0.46
Firmicutes; Erysipelotrichi; Erysipelotrichales; Erysipelotrichaceae; Turicibacter	0.03 %	0.04 %	**0.01 %**	1.39	0.21
Firmicutes; Erysipelotrichia; Erysipelotrichales; Erysipelotrichaceae; Catenibacterium	0.02 %	0.02 %	**0.08 %**	0.86	3.31
Firmicutes; Negativicutes; Selenomonadales; Veillonellaceae; Dialister	1.05 %	**0.34 %**	1.75 %	0.32	1.67
Proteobacteria; Deltaproteobacteria; Desulfovibrionales; Desulfovibrionaceae; Bilophila	0.04 %	**0.08 %**	0.07 %	2.34	2.03

Complete list of significant genus level changes post-intervention compared to the baseline. FDR corrected p-values >0.05 are show in bold
