# Supplementary material for: A low FODMAP diet is associated with changes in the microbiota and reduction in breath hydrogen but not colonic volume in healthy subjects
Source: PLoS One. 2018 Jul 26;13(7):e0201410. doi: 10.1371/journal.pone.0201410 (PMC6062106; doi:10.1371/journal.pone.0201410)
Supplement: S4 Table — Short chain fatty acid concentrations per study group. MD, maltodextrin; OF, oligofructose; BL, baseline; PI, post-intervention. No significant differences between the groups. Values shown are mean (SD) μmol/g wet stool per group. (PDF) [file pone.0201410.s008.pdf]

S4 Table. Faecal short chain fatty acid concentrations.

	Acetic	Propanoic	Isobutyric	Butyric	Isovaleric	Valeric
MD-BL	24.0 (4.1)	6.6 (3.4)	1.3 (0.4)	8.9 (6.2)	1.4 (0.5)	1.4 (0.5)
MD-PI	23.0 (3.1)	6.2 (3.1)	1.3 (0.5)	8.4 (6.9)	1.6 (0.7)	1.3 (0.7)
OF-BL	25.6 (3.8)	9.1 (5.7)	1.4 (0.7)	12.5 (8.6)	1.6 (0.8)	2.0 (1.4)
OF-PI	24.0 (3.1)	6.4 (3.6)	1.1 (0.4)	8.6 (6.2)	1.2 (0.5)	1.2 (0.6)

Short chain fatty acid concentrations per study group. MD, maltodextrin; OF, oligofructose; BL, baseline; PI, post-intervention. No significant differences between the groups. Values shown are mean (SD) µmol/g wet stool per group.
