# Supplementary material for: A low FODMAP diet is associated with changes in the microbiota and reduction in breath hydrogen but not colonic volume in healthy subjects
Source: PLoS One. 2018 Jul 26;13(7):e0201410. doi: 10.1371/journal.pone.0201410 (PMC6062106; doi:10.1371/journal.pone.0201410)
Supplement: S5 Table — Statistically significant associations between genus level taxa and short chain fatty acids, across both time points, showing positive or negative associations calculated with linear mixed models. (PDF) [file pone.0201410.s009.pdf]

S5 Table. Associations between the microbiota and short chain fatty acids.

Genus level taxon	Acetic	Butyric	Propanoic	Isobutyric	Isovaleric	Valeric
Actinobacteria; Actinobacteria; Micrococcales; Micrococcaceae; Rothia			-	-	-	-
Actinobacteria; Coriobacteriia; Coriobacteriales; Coriobacteriaceae; Adlercreutzia	-	-	-			-
Actinobacteria; Coriobacteriia; Coriobacteriales; Coriobacteriaceae; Eggerthella	-					
Actinobacteria; Coriobacteriia; Coriobacteriales; Coriobacteriaceae; uncultured	-	-	-			
Bacteroidetes; Bacteroidia; Bacteroidales; Porphyromonadaceae; Butyricimonas	-		-			
Bacteroidetes; Bacteroidia; Bacteroidales; Porphyromonadaceae; Odoribacter	-		-			-
Bacteroidetes; Bacteroidia; Bacteroidales; Porphyromonadaceae; Parabacteroides		-				
Bacteroidetes; Bacteroidia; Bacteroidales; Rikenellaceae; Alistipes		-				
Firmicutes; Clostridia; Clostridiales; Christensenellaceae; Christensenella		-				
Firmicutes; Clostridia; Clostridiales; FamilyXIIIIncertaeSedis; IncertaeSedis	-	-				
Firmicutes; Clostridia; Clostridiales; Lachnospiraceae; Anaerostipes				-	-	
Firmicutes; Clostridia; Clostridiales; Lachnospiraceae; Roseburia	+	+				
Firmicutes; Clostridia; Clostridiales; Ruminococcaceae; Anaerofilum					+	
Firmicutes; Clostridia; Clostridiales; Ruminococcaceae; Flavonifractor	-				+	
Firmicutes; Clostridia; Clostridiales; Ruminococcaceae; Ruminococcus				+	+	
Firmicutes; Erysipelotrichia; Erysipelotrichales; Erysipelotrichaceae; uncultured	-					
Firmicutes; Negativicutes; Selenomonadales; Acidaminococcaceae; Acidaminococcus	+		+			+
Firmicutes; Negativicutes; Selenomonadales; Acidaminococcaceae; Succiniclasticum	+	+				
Firmicutes; Negativicutes; Selenomonadales; Veillonellaceae; Veillonella				-	-	
Proteobacteria; Gammaproteobacteria; Pasteurellales; Pasteurellaceae; Haemophilus				-	-	
Verrucomicrobia; Verrucomicrobiae; Verrucomicrobiales; Verrucomicrobiaceae; Akkermansia		-				

Statistically significant associations between genus level taxa and short chain fatty acids, across both time points, showing positive or negative associations calculated with linear mixed models
