# Supplementary material for: A low FODMAP diet is associated with changes in the microbiota and reduction in breath hydrogen but not colonic volume in healthy subjects
Source: PLoS One. 2018 Jul 26;13(7):e0201410. doi: 10.1371/journal.pone.0201410 (PMC6062106; doi:10.1371/journal.pone.0201410)
Supplement: S1 Methods — Detailed description of methods for dietary and metabolomics analysis. (PDF) [file pone.0201410.s010.pdf]

Supplementary methods

Calculation of FODMAP intake

FODMAP intake was estimated using a FODMAP database produced by King's College London based on published literature[1-5].

In brief, for lactose and free fructose, McCance and Widdowson data was used[6]. For fructans, galacto-oligosaccharides, sorbitol and mannitol published data for individual foods was used to provide data for the appropriate foods in the database[1-5]. Foods with published data e.g. apple, onion, wheat based bread, wheat based pasta were added to the database. For foods where only qualitative data was available, e.g. those known to be low in FODMAPs e.g. meat, fish it was assumed that they contributed no FODMAPs. For foods known to contain FODMAPs without any published data, data for similar foods were used e.g. data for kidney beans and lima beans was used for other similar pulses. For composite dishes and recipes, the raw ingredients used to make the composite meal were used to calculate the FODMAP levels for that composite dish or recipe taking into considerations effects that cooking would have on water content for each ingredient. The database that was developed contained estimated levels of FODMAPs for over 90% of foods in the database.

Automated gas analysis

Colonic gas was identified as described previously[7,8], with quantification through an automated process supplemented by operator verification of gas pockets.

Automated gas analysis found little or no gas in the colon. Visual inspection of scans by an experienced operator (SP) found that only small gas pockets could be

identified in a small number of subjects, and that manual analysis was unlikely to identify significant amounts of gas or changes with intervention.

Urinary metabolomics

Sample Preparation: thawed urine samples of subjects were centrifuged at 10,000 *g* for 10 min and the supernatant was diluted 1:2 urine:water in HPLC amber glass vials containing 200 µL glass inserts. Blank was prepared the same without using urine. For metabolomics analysis, pooled QC sample was prepared by mixing 20 µL aliquots taken from each urine sample and was treated the same as described for the samples.

Urine analysis: liquid chromatography-high resolution mass spectrometry (LC-HRMS) was performed on a BEH HILIC column (2.1 x 100 mm, 1.7 µm particle size; Waters, Milford, USA) using Accela UHPLC system coupled to orbital trap mass spectrometer (Exactive-Orbitrap MS, Thermo Fisher Scientific, Bremen, Germany). Mobile phases A: 50:50 and B: 95:5 acetonitrile:ammonium acetate (10 mM) were used with a flow rate of 400 µL/min. 5 µL sample was injected in a gradient of 1% A and increased over 12 min to 100% A then the composition was returned to 1% A over 3 min. The column was kept at 40 °C and samples at 4 °C during the analysis. LC-HRMS datasets were acquired simultaneously in full scan mode with a resolution of 25,000 at a scan rate of 4 Hz from *m/z* 60-1000. The MS parameters were: spray voltage 3.2 kV (ESI+), 2.4 kV (ESI-), capillary voltage 25 V (ESI+), -27 V (ESI-). Sheath, auxiliary and sweep gas flow rates were: 20, 5 and 5 (arbitrary unit), respectively. Capillary and heater temperature were maintained at 350 °C and 120 °C, respectively, for both modes. For metabolomics analysis, all urine samples were

randomised and analysed in a single analytical run with pooled QC urine sample interspaced throughout the run.

Data analysis: Urine Datasets were pre-processed using Progenesis QI (Nonlinear Dynamics, Waters, Durham, USA). Multivariate analysis using principal component analysis (PCA) and orthogonal partial least squares-discriminant analysis (OPLS-DA) were used to investigate urinary metabolic changes using Simca P +14 (MKS, Umeå, Sweden).

Each peak was assigned a number of potential identifications from the Human Metabolome Database (HMDB) [9]. HMDB identifications were filtered by those with a corresponding match in the Kyoto Encyclopedia of Genes and Genomes (KEGG) database for compounds in metabolic pathways [10]. Peak intensities were aggregated for all HMDB identifications within a given metabolite superclass, such as 'Lipids' or 'Amino Acids', to give an aggregate metabolite score. Change in aggregate metabolite score could then be calculated from baseline for each of the study groups, MD or OF.

Faecal short chain fatty acid (SCFA) analysis

Sample preparation: After thawing of the faecal sample, 0.5 g was suspended in 5 ml 0.5 % phosphoric acid (H3PO4). The samples were then vortexed for 1 min and centrifuged at 25,000 g for 10 min. The SCFA-containing supernatant was filtered through cellulose acetate membrane with pore size 0.2 um. 700 uL of this supernatant was mixed with 700 uL of ethyl acetate, vortexed for 30 s and then centrifuged at 25,000 g for 10 min. 300 uL of the supernatant (ethyl acetate layer) was transferred to a clean eppendorf and stored at -80°C until analysis.

Quantification of SCFA by GC–MS: Short chain fatty acid (SCFA) quantification was carried out by gas chromatography–mass spectrometry (GC-MS). Separation and detection of the short chain fatty acids of interest was achieved with splitless injection of the ethyl acetate extract (1 µl) using a Trace GC Ultra (Thermo Scientific, Manchester, UK) coupled with a DSQII mass spectrometer (Thermo Scientific). The inlet temperature was 200 °C and data acquisition was started at 5 min, positive ion mode, in full scan mode with a mass range of m/z 30–250, scan rate 500 amu s−1. Separations were performed on a Zebron ZB-FFAP column (length 30 m, inner diameter 0.25 mm, and film thickness 0.25 µm; Phenomenex Inc., Macclesfield, UK). The initial oven temperature was set at 60 °C for 1 min then increased at 8 °C min−1 to 180 °C. Compound identification was achieved by matching with database mass spectra (NIST/EPA/NIH Mass Spectral Library, Version 2.0d, NIST, Gaithersburg, MD, USA). Identification was further verified by comparing with the retention times and mass spectra of authentic standards. Concentrations of analyte were calculated using 'Xcalibur' software (Thermo Scientific, UK). Retention time and specific ions used for quantification are detailed below for each analyte: acetic acid (7.10 min, m/z 60); formic acid (8.17 min, m/z 46); propanoic acid (8.41 min, m/z 57); isobutyric acid (8.83 min, m/z 73); butyric acid (9.75 min, m/z 88); valeric acid (11.35 min, m/z 73).

References

1. Whelan K, Abrahmsohn O, David GJ, et al. Fructan content of commonly consumed wheat, rye and gluten-free breads. *International journal of food sciences and nutrition* 2011;62(5):498-503. doi: 10.3109/09637486.2011.553588 [published Online First: 2011/03/23]
2. Muir JG, Shepherd SJ, Rosella O, et al. Fructan and free fructose content of common Australian vegetables and fruit. *Journal of agricultural and food chemistry* 2007;55(16):6619-27. doi: 10.1021/jf070623x [published Online First: 2007/07/11]
3. Muir JG, Rose R, Rosella O, et al. Measurement of short-chain carbohydrates in common Australian vegetables and fruits by high-performance liquid chromatography (HPLC). *Journal of agricultural and food chemistry* 2009;57(2):554-65. doi: 10.1021/jf802700e
4. Biesiekierski JR, Rosella O, Rose R, et al. Quantification of fructans, galacto-oligosacharides and other short-chain carbohydrates in processed grains and cereals. *J Hum Nutr Diet* 2011;24(2):154-76. doi: 10.1111/j.1365-277X.2010.01139.x [published Online First: 2011/02/21]
5. Yao CK, Tan HL, van Langenberg DR, et al. Dietary sorbitol and mannitol: food content and distinct absorption patterns between healthy individuals and patients with irritable bowel syndrome. *J Hum Nutr Diet* 2014;27 Suppl 2:263-75. doi: 10.1111/jhn.12144 [published Online First: 2013/08/03]
6. McCance and Widdowson's The Composition of Foods: Seventh Summary Edition: The Royal Society of Chemistry 2015:P001-630.
7. Murray K, Wilkinson-Smith V, Hoad C, et al. Differential effects of FODMAPs (fermentable oligo-, di-, mono-saccharides and polyols) on small and large intestinal contents in healthy subjects shown by MRI. *Am J Gastroenterol* 2014;109(1):110-9. doi: 10.1038/ajg.2013.386 [published Online First: 2013/11/20]
8. Major G, Pritchard S, Murray K, et al. Colon Hypersensitivity to Distension, Rather Than Excessive Gas Production, Produces Carbohydrate-related Symptoms in Individuals with Irritable Bowel Syndrome. *Gastroenterology* 2016 doi: 10.1053/j.gastro.2016.09.062 [published Online First: 2016/10/18]
9. Wishart DS, Jewison T, Guo AC, et al. HMDB 3.0--The Human Metabolome Database in 2013. *Nucleic Acids Res* 2013;41(Database issue):D801-7. doi: 10.1093/nar/gks1065 [published Online First: 2012/11/17]
10. Kanehisa M, Furumichi M, Tanabe M, et al. KEGG: new perspectives on genomes, pathways, diseases and drugs. *Nucleic Acids Res* 2017;45(D1):D353-D61. doi: 10.1093/nar/gkw1092 [published Online First: 2016/11/28]
