# Supplementary material for: A low FODMAP diet is associated with changes in the microbiota and reduction in breath hydrogen but not colonic volume in healthy subjects
Source: PLoS One. 2018 Jul 26;13(7):e0201410. doi: 10.1371/journal.pone.0201410 (PMC6062106; doi:10.1371/journal.pone.0201410)
Supplement: S1 Protocol — (PDF) [file pone.0201410.s011.pdf]

DETAILED STUDY PROTOCOL

TITLE

A randomised controlled trial of the effect of the low FODMAP diet and dietary oligofructose on gastrointestinal form, function and microbiota in healthy volunteers: FOG

Version 1.0 16th July 2014

BACKGROUND AND RATIONALE

Oligofructose (OF) is a fructose-based oligosaccharide and defined in the EU as a dietary fibre. Enzymatically derived from the longer chain inulin, extracted from chicory, it is commonly used in processed food to improve mouth feel in fat-free products. OF is poorly digested and absorbed in the small bowel so passes to the colon where it is fermented by the bacteria usually resident in the colon, termed the microbiota. Its presence in the colon alters the composition of the microbiota, with reported potential benefits to health, leading to its description as a 'prebiotic'[1].

Recently, however, such poorly digested carbohydrates grouped together by the term FODMAP (fermentable oligo-, di-, mono-saccharides and polyols) have been proposed to exacerbate symptoms of irritable bowel syndrome (IBS) such as abdominal discomfort and bloating. Dietary exclusion of foods containing FODMAPs, such as wheat, dairy and certain fruit and vegetables, has been proposed as a treatment for IBS, with some evidence to support this[2-4]. FODMAPs are thought to induce symptoms either by drawing water into the small bowel by osmosis, or through gaseous distension of the large bowel, or a combination of these combined with metabolite effects on motility.

The metabolic products of bacterial fermentation are short-chain fatty acids (SCFAs) and gases, such as hydrogen and methane. All of these can become substrates for growth of other bacteria, leading to a complex ecosystem. The SCFA butyrate provides nutrition to colonic epithelial cells, a phenomenon that has created interest in the role of butyrate, and the microbiota, in colon cancer, inflammatory bowel disease and functional gastrointestinal disorders. Recently butyrate has been shown to influence the development of colonic neurons, with potential effects on colonic motility and transit of luminal contents through the bowel[5]. Thus, there appears to be a potential interaction between the colonic microbiota, complex carbohydrates in the diet and gastrointestinal transit with the suggestion of a possible effect on luminal serotonin levels[6]. It is currently unclear how long a change in diet takes to alter the microbiota, but changes have been observed within a day of radical alteration in carbohydrate content of the diet[7].

Nottingham has world-leading expertise in Magnetic Resonance Imaging (MRI), particularly of in-body, non-invasive imaging of food transit and conformational change through the

gastrointestinal tract in humans. MRI imaging is also widely used to measure organ volumes. Within research conducted during studies of IBS patients (06GM006) and on the mode of action of Moviprep (10/H0906/50), the University of Nottingham has recently developed protocols to measure regional colonic volumes from coronal dual echo Fast Field imaging as briefly illustrated in the figure below[8]. It is also possible to measure separately the volume of intraluminal gas (as it provides no signal) while the chyme usually gives a bright signal[9].

With MRC funding (NREC approval 11/EM/0245) the UoN GI MRI research group has also recently completed validation of a measure of whole gut transit time using MRI localisation of Gadolinium-filled capsules made of polyoxymethylene, a biologically inert substance[10]

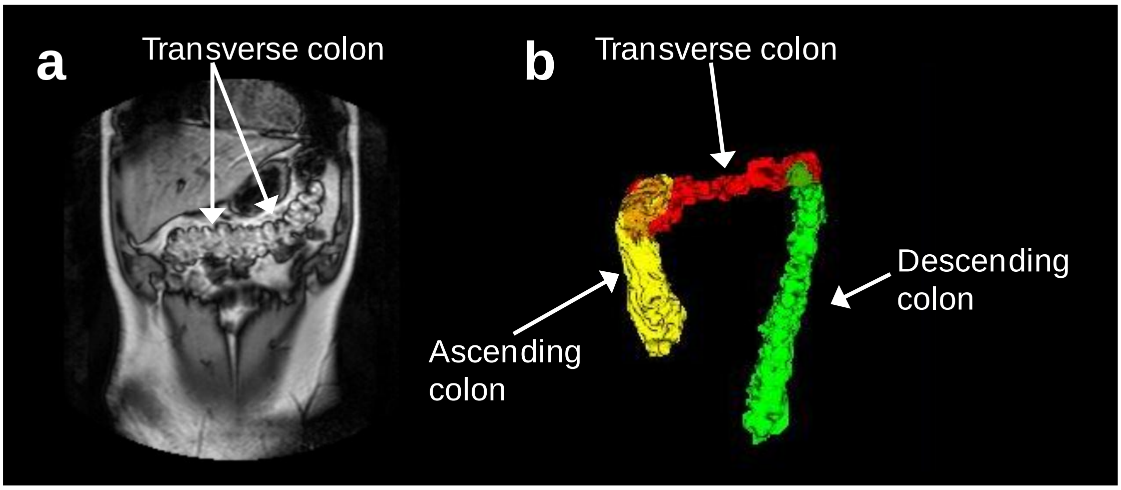

This panel shows (a) one coronal 'dual FFE' MRI image of the transverse colon of a healthy volunteer taken at fasted baseline. (b) a three-dimensional reconstruction of the different regions (ascending, transverse and descending) of the colon of another healthy volunteer.

Last year we combined these two techniques to gather pilot data on the effects of dietary OF and the longer chain polysaccharide from which it is derived, inulin, on colonic physiology. This pilot work found an 18% increase (p=0.02) in fasting colonic volume after dietary supplementation with OF (abstract BSG 2014). This could not be accounted for by MRI measured changes in gas but breath hydrogen also rose significantly, suggesting increased levels of fermentation. One unifying hypothesis would be that increased nutrient availability leads to bacterial proliferation, increasing the mass of microbiota present. Whole gut transit time was slower, without reaching significance. This was the opposite of what we expected but is biologically plausible if delay in colonic transit maximises the opportunity for nutrient recovery.

The previous study was only a pilot study and as such had a few imperfections: it was uncontrolled with no open label and diet was not standardised between participants. We now wish to test our hypothesis that increased dietary content of poorly digested carbohydrates increases colonic volume in healthy volunteers. We will also test whether such changes can be explained by colonic gas volume measured, slowing of whole gut transit time or increased metabolic activity of the microbiota. We will also gather samples to allow exploratory work on how these dietary changes affect the urinary metabolome and, if future funding allows, changes in the microbiota.

TRIAL OBJECTIVES AND PURPOSE

Purpose

The purpose of the study is to investigate the effect of altering the amount of poorly digested carbohydrate in the diet for a week on the fasting colonic volume in healthy volunteers, and to explore possible mechanisms that underlie any differences found.

Primary Objective

The primary objective is to determine the change from baseline in fasting colonic volume in healthy volunteers who follow a diet low in fermentable carbohydrates (the low FODMAP diet) to which carbohydrates are then added back as supplements, and to assess whether different carbohydrate supplements can alter the change measured.

Secondary Objectives

Secondary objectives include:

- Examining factors that might explain changes in volume, including speed of whole gut transit time, colonic gas volume and degree of fermentation in the colon
- Exploring other effects of dietary change on the metabolic products of the microbiota

Hypotheses

- The increase in fasting colonic volume measured by MRI will be greater in participants who supplement a low FODMAP diet with OF than in those who supplement with maltodextrin
- The Weighted Average Position Score[10] of MRI transit markers 24 hours after ingestion will be no different between the two groups
- The increase in fasting colonic gas volume measured by MRI will be no different between the two groups
- Supplementation with OF will lead to a greater increase in fasting breath hydrogen than will supplementation with maltodextrin

DETAILS OF PRODUCTS

The intervention to be tested in this study is dietary intake of fermentable carbohydates. The assessments made will not be tests of efficacy but of effects on physiological performance. The products used in this study are Generally Regarded As Safe (GRAS) and are available for general purchase.

Description

A. Maltodextrin, 7 gram serving taken 2 times daily for 7 days. Maltodextrin is a long-chain glucose polysaccharide, marketed by the Hut group, (Northwich, UK). The Material Safety Data Sheet for Maltodextrin lists no known side effects.

B. Oligofructose, 7 gram serving taken 2 times daily for 7 days. Oligofructose as Orafti P95® is manufactured, marketed and sold in the EU by Beneo, Germany. It is not considered to have any side effects in the doses proposed.

Both products will have a small amount of Carmine Red food dye - E120 - added to the supplement container. This will be approximately 5 grams of red powder in approximately 200 grams of supplement. Carmine Red is not absorbed by the bowel and has no side effects. It is used as a marker of whole gut transit. Its addition to the supplement will improve masking by making both powders pink, and will aid compliance. Carmine Red is of animal origin and not suitable for vegetarians. A similar process was approved by the University of Nottingham Medical School research ethics committee for the 2013 pilot study (A11072013 SCS MRI).

Packaging and labelling

Both products will be packaged into opaque plastic containers in the NDDC in a food preparation area by staff suitably trained in food hygiene. A suitably calibrated plastic scoop will be provided. Labelling will done by an independent member of NDDC or SPMMRC staff not otherwise involved in the study who will have access to the randomisation codes.

Storage, dispensing and return

Both products can be stored at room temperature and will be kept in the NDDC until they are dispensed. Before dispensing and on return containers will be weighed to assess the amount of study product used to allow a calculation of compliance. After return both products are safe to be disposed of in general waste.

Known Side Effects

Both products are recognised as food additives and are not associated with side effects. More information on Orafti P95® is available at www.beneo.com.

STUDY DESIGN

Trial Configuration

This will be a parallel group, double- blind, randomized controlled trial

Primary endpoint

- Percentage change in fasting colonic volume after one week of dietary intervention as measured by MRI

Secondary endpoints

- Change in whole gut transit time (WGTT) as determined by Weighted Average Position Score (WAPS) of MRI transit markers 24 hours after ingestion, after one week of dietary intervention
- Percentage change in fasting colonic gas volume after one week of dietary intervention as measured by MRI
- Change in fasting breath hydrogen concentration after one week of dietary intervention, measured in parts per million
- Change in fasting breath methane concentration after one week of dietary intervention, measured in parts per million

Exploratory endpoints

Biological samples will be collected to allow analysis of the following exploratory endpoints once additional funding has been confirmed.

- Change in urinary metabolite concentrations after one week of dietary intervention
- Change in faecal microbiota concentrations after one week of dietary intervention
- Change in faecal short-chain fatty acid concentrations after one week of dietary intervention

Stopping rules and discontinuation

Given the short time frame of the study there will be no planned stopping rules.

Randomisation and Blinding

Participants will be allocated to a treatment group according to a randomization schedule generated by an independent member of staff at the SPMMRC or NDDC using the online program www.randomization.com. Randomization will be 1:1 in blocks of 6 to prevent imbalance between groups.

Participants, investigators and analysts will be blind to treatment allocation until analysis of the primary and secondary endpoints is complete. The intervention supplements are both white powders which are indistinguishable. They will be supplied in opaque containers with the gross weight recorded on the side. Labelling will be completed by members of NDDC/ SPMMRC staff not directly involved with the study.

The code will be kept by the Chief Investigator in a sealed envelope and on a password

protected computer not accessible by the study team. No adverse events directly relating to the study product are expected. Should a serious adverse event occur the intervention will be discontinued without any need to unblind as the supplement will have no bearing on subsequent management.

Trial Management

The trial is not sufficiently big to require a trial Steering Committee or Data Monitoring Committee. The Chief Investigator has overall responsibility for the study and shall oversee all study management.

The data custodian will be the Chief Investigator.

Duration of the Trial and Participant Involvement

End of the trial

The trial is expected to last up to 4 months from initiation to the last visit of the last patient.

The last visit of the last patient will be considered to be the end of the trial. Laboratory and data analysis will continue after this time.

Selection and withdrawal of participants

Recruitment

Healthy volunteers who meet eligibility criteria will be recruited by general advertisement on Nottingham University campuses and through the SPMMRC and NDDC databases of healthy volunteers who have expressed a wish to be contacted about further studies. We will approach other university departments e.g. MRC Institute of Hearing Research to ask them to assist with advertising. We will also advertise through our social media presence on Facebook and Twitter e.g. www.facebook.com/UoN.Gastrointestinal.MRI.

Prospective participants will have the study fully explained and will be given an information sheet to read and retain. It will be explained to the potential participant that entry into the trial is entirely voluntary and that they can withdraw at any time but attempts will be made to avoid this occurrence. In the event of their withdrawal it will be explained that their data collected so far cannot be erased and we will seek consent to use the data in the final analyses where appropriate.

The limited resources of the trial mean that we will not recruit participants who do not understand English. Other exclusions will include those for whom a low FODMAP diet or the supplement would prove difficult because of medical issues or ethical choices e.g. diabetes, veganism.

Eligibility Criteria

Inclusion

- Aged 18 or older
- Able to give informed consent

Exclusion

- Self-declared vegetarian, vegan or kosher/ halal diet who cannot eat carmine red dye
- Pregnancy declared by candidate
- History declared by the candidate of pre-existing gastrointestinal disorder that may affect bowel function
- A positive diagnosis of irritable bowel syndrome based on the Rome III criteria questionnaire
- Reported history of previous resection of the oesophagus, stomach or intestine (excluding appendix)
- Intestinal stoma
- Any medical condition making participation potentially compromising participation in the study e.g. diabetes mellitus, respiratory disease limiting ability to lie in the scanner
- Contraindications for MRI scanning i.e. metallic implants, pacemakers, history of metallic foreign body in eye(s) and penetrating eye injury
- Will not limit alcohol intake to ≤ 35 units/ week and ≤ 8 units per day during trial
- Unable to stop drugs known to alter GI motility including mebeverine, opiates, monoamine oxidase inhibitors, phenothiazines, benzodiazepines, calcium channel antagonists for the duration of the study (Selective serotonin reuptake inhibitors and low dose tricyclic antidepressants will be recorded but will not be an exclusion criteria)
- Antibiotic or prescribed probiotic treatment in the past 8 weeks
- Inability to lie flat or exceed scanner limits of weight <120kg
- Poor understanding of English language
- Participation in night shift work the week prior to the study day. Night work is defined as working between midnight and 6.00 AM
- Participation in any medical trials for the past 3 months
- Anyone who in the opinion of the investigator is unlikely to be able to comply with the protocol e.g. cognitive dysfunction, chaotic lifestyle related to substance abuse

Expected duration of participant participation

Each participant will be involved for a minimum of 16 days from enrolment. This may be extended, either for patient convenience or to accommodate bookings on the MRI scanner, with a gap between consent and commencement of the first food diary.

Removal of participants from therapy or assessments

Participants may be withdrawn from the trial either at their own request or at the discretion of the Investigator. The participants will be made aware that this will not affect their future care. Participants will be made aware (via the information sheet and consent form) that should

they withdraw the data collected to date cannot be erased and may still be used in the final analysis.

Informed consent

All participants will provide written informed consent. The Informed Consent Form will be signed and dated by the participant before they enter the trial. The Investigator will explain the details of the trial and provide a Participant Information Sheet, ensuring that the participant has sufficient time to consider participating or not. The Investigator will answer any questions that the participant has concerning study participation. Where consent is obtained by a student investigator they will be directly supervised in the process until the PI is assured of their competence, and then indirectly supervised with help available.

Informed consent will be collected from each participant before they undergo any interventions (including physical examination and history taking) related to the study. One copy of this will be kept by the participant, one by the Investigator, and a third by the SPMMRC.

Should there be any subsequent amendment to the final protocol, which might affect a participant's participation in the trial, continuing consent will be obtained using an amended Consent form which will be signed by the participant.

TRIAL TREATMENT AND REGIMEN

Study sites

Nottingham Digestive Diseases Centre (NDDC), Queen's Medical Centre
Sir Peter Mansfield Magnetic Resonance Centre (SPMMRC), University Park Campus

Study Schematic

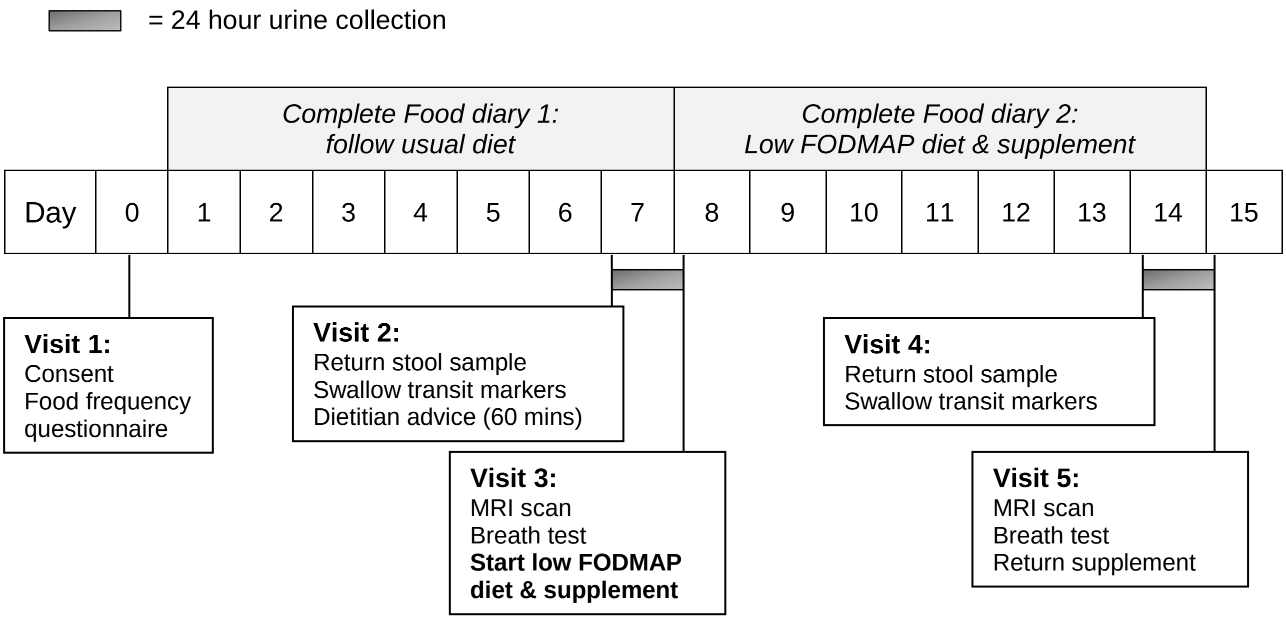

Details of Trial Regimen

This study will use a parallel group design with 18 subjects per group.

Visit 1 (Consent) – Day 0
Visit 1 will take place at the NDDC and last 20-30 minutes. After consent has been obtained participants will be screened against eligibility criteria will be enrolled if they are eligible. An MRI safety screening questionnaire will be completed as part of this process. A record will be taken of height, weight, current medication and any medical conditions and smoking history.

Participants will then fill in a food frequency questionnaire, the Comprehensive Nutrition Assessment Questionnaire (CNAQ), a questionnaire validated in an antipodean population to assess FODMAP content of the diet. This is pilot work to look at the utility of the CNAQ in a UK population.

Participants will then be instructed in how to complete a one week food diary. The first day of the food diary will be designated Day 1. They will also be told how to collect a stool sample.

They will be given to take away:

- A one week food diary based on the EPIC food diary, with guidance
- A stool collection kit including gloves, a cardboard tray and sample pots, with instructions

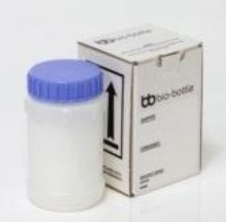

The stool collection kit has been used extensively in other departmental studies. A microbiology container that meets UN3373B standards for transport (Biobottle™; see picture) will be provided, along with a freezable gel pack. Participants will be asked to freeze the gel pack and collect 3 cherry-sized aliquots of stool from a single motion. This should be done at some point in the two days before Visit 2. Once collected, sample tubes can be put in the Bio-Bottle™ along with the gel pack and stored in the home freezer until visit 2.

Participants should not take excessive alcohol during the study and should abstain from alcohol from 8am on Day 6 (48 hours before the MRI scan).

Visit 2 – Day 7

Visit 2 will take place in the NDDC and last around 90 minutes. Participants will attend around 8.30am, and will fast until they attend except for a small amount of water with essential tablets. They will return their stool samples, which will be immediately transferred to the -80C freezers of the NDDC. They will provide a urine sample which will be spun and frozen. Having emptied their bladders, participants will be asked to start a 24 hour urine collection.

They will then be offered a standard breakfast with water and be asked to swallow 5 transit marker pills. The marker pills (see picture) are made of polyoxymethylene and are the shape

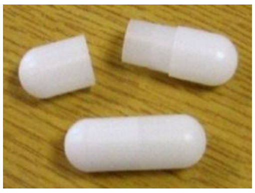

of a size 1 gelatine capsule (20mm by 7mm). They are filled with 0.4 ml of 15µM Gadoteric acid solution and sealed with cyanoacrylate superglue. They have previously been used in studies with healthy volunteers and patients without complication (NRES approvals 10/H0906/50 and 11/EM/0245).

Note that up to 6 participants will attend visit 2 at the same time. 3 will take their marker pills between 8.30 and 9.30, and 3 will take them between 9.30 and 10.30 in accordance with the order of MRI scanning the following day.

During the visit participants will take part in a group session of advice on how to follow the low FODMAP diet. This will be led by Ms Krishnasamy, a registered dietitan trained in the instruction of the low FODMAP diet. Written material, published by Guy's and St Thomas' NHS Foundation trust, will be provided as it would be in clinical practice, on loan. This includes 2 booklets: "Reducing Fermentable Carbohydrates the low FODMAP way" and "Suitable Products for the low FODMAP diet". *Note that participants will not start to follow the low FODMAP diet until after their MRI scan the next morning.*

At the end of the visit participants will take away:

- A 24-hour urine collection bottle containing boric acid, with equipment to allow collection
- A standard packed lunch
- A standard meal for evening consumption

Participants may snack in addition to the meals provided but should use the food supplied for their main meals. They should continue their food diaries until the next morning. After 10pm that night no food should be eaten and only water should be drunk.

Visit 3 – Day 8
Visit 3 will take place at the 1.5T scanner of the SPMMRC and will last up to 1 hour. Participants should attend at 8am (first scan session) or 9am (second scan session). They should not eat or drink after waking, except a small sip of water for essential tablets.

Participants will return their 24 hour urine collection. This will be weighed and aliquots taken for metabolomic analysis. They will also return their food diary. During their visit participants will undergo 2 procedures:

- A test of breath hydrogen and methane using the GastroCHeck meter (Bedfont, UK)
- A sequence of MRI scans (15-20 minutes)

The exact order of procedures may vary. Prior to the MRI scans participants will change into surgical scrubs (provided) and confirm the MRI safety screening questionnaire. The MRI scan sequences will measure colonic volume, colonic gas volume and other MRI parameters (T_1,T_2) that describe physico-chemical characteristics of the bowel and its contents.

After all procedures have been completed participants will be given a low FODMAP breakfast and their study supplement. The supplement will be either/ or:

A. Maltodextrin (the Hut Group, Northwich)
B. Oligofructose as OraftiP95® (Beneo, Germany)

Participant should take 7 grams (= one scoop) of supplement twice daily for a week. The supplement should be added to a warm/ hot drink and stirred. Participants will take their first serving of study supplement at the SPMMRC to ensure there are no issues with preparation or consumption. Participants should follow a low FODMAP diet until their next MRI scan.

Participants will take away from the visit:
- A second food diary to complete over the next week
- A second stool collection kit

Days 8 – 13
During days 8 – 13 participants should adhere to the low FODMAP diet. The study team will attempt to contact participants at least once during that time to discuss any issues arising and offer encouragement, and participants will be free to contact the study team with any issues that arise.

Participants should collect a second set of stool samples during the two days before Visit 4, in the same manner as described before Visit 2. They should abstain from alcohol from 8am on Day 13 (48 hours before the MRI scan).

Visit 4 – Day 14
Visit 4 will take place at the NDDC and last 15 - 20 minutes. As for Visit 2, participants should fast until they attend around 8.30am. Stool and urine samples will be collected. Participants can return their low FODMAP advice booklets at this visit or Visit 5. The food diary should be continued until Visit 5.

Participants will be provided with a low FODMAP breakfast and asked to swallow 5 transit

markers. They should continue to take their supplement for the rest of the day. The last serving of supplement should be the second serving on Day 14.

At the end of the visit participants will take away:

- A 24-hour urine collection bottle containing boric acid, with equipment to allow collection
- A low FODMAP packed lunch
- A low FODMAP meal for evening consumption

Participants may snack in addition to the meals provided but should use the food supplied for their main meals. They should continue their food diaries until the next morning. After 10pm that night no food should be eaten and only water should be drunk.

Visit 5 – Day 15

Visit 5 will take place at the 1.5T scanner of the SPMMRC and will last up to 1 hour. As for Visit 3 participants should attend at 8am (first scan session) or 9am (second scan session). They should not eat or drink after waking, except a small sip of water for essential tablets.

They will return their second urine collection and food diary, along with any outstanding information booklets. They will also return their supplement container to allow weighing for compliance.

Participants will then undergo the same procedures as for Visit 3: breath testing and MRI scanning. The completion of these procedures will be the end of the participant's involvement in the trial. They will be offered a light snack and a drink.

Compliance

Supplement compliance will be assessed by measuring the gross weight of the supplement container at the start and end of the week. Compliance will be calculated as (actual weight change / expected weight change) * 100%, with an acceptable range of 70 – 140%.

Dietary compliance will be assessed by food diary. No specific compliance parameters will be pre-specified but FODMAP intake will be compared between groups. To allow for tail-off and omission in reporting analysis will be done on the four days with highest calorie intake.

Transport and Storage of Tissues

Samples will be stored in linked anonymised format at the NDDC and labelled using a combination of date of birth, initials and study number to permit accurate linkage to the case report form and consent form.

Samples will be stored in -80C freezers. Essential maintenance of the laboratory space and equipment is performed by two laboratory technicians. The master database will be held by the Chief Investigator in a password encrypted file.

Once analysis has taken place, samples will be stored within the Research Tissue Bank for future research (DI Prof Jim Lowe- Licence Number 12265) if participants are agreeable and sign the optional clause on the consent form. Where participants do not agree to the future use of the samples they will be destroyed in accordance with the Human Tissue Act, 2004.

Laboratory Analysis

MRI Scanning Schedule

Subjects will be scanned on a research dedicated 1.5T Philips Achieva MRI scanner, using a parallel imaging SENSE 16-element torso coil. A range of MRI sequences will be used to image the abdomen including:

1) A T1 weighted 3D turbo field echo (TFE) sequence (repetition time (TR) = 4.0 ms, echo time (TE) = 1.9 ms, flip angle (FA) 10°, acquired resolution (AQR) 2.3 x 2.3 x 5 mm^3) will be used to locate and count the number of transit marker pills remaining in the colon at 24 and 48 hours. A multi shot T1 weighted TFE sequence with fat suppression (TR = 2.7 ms, TE = 1.4, AQR 1.8 x 2.6 x 3.6 mm^3, sensitivity encoding (SENSE) factor = 2.0) will be used to create a movie which will allow colonic rotation and give 3D visualisation of the colon. This will further clarify the location of the marker pills.

2) A high resolution bTFE sequence to acquire images of the contents of the ascending colon (TR = 3.1 ms, TE = 1.56 ms, acquired resolution = 1.50 mm x 1.50 mm, reconstructed resolution= 0.86 x 0.86, 8 slices 5 mm thick, up to 5.0 mm gap to cover whole ascending colon, flip angle 45°).

3) We will assess T_1/ T_2 of colonic contents using previously published methods[11-13].

Depending on the sequence, each image set will be acquired with an expiration breath-hold between 13 and 24 seconds, monitored using a respiratory belt. Subjects will spend approximately 15 minutes inside the magnet.

Whole gut transit

This will follow the method described in Chaddock et al.[10] Transit will be assessed and scored from the scans performed 24 hours after marker ingestion. The previously documented scoring system divides the bowel into 8 sections, (Figure 1A) allowing each capsule to be scored according to its position in the bowel after 24 hours[10]. A weighted average position score of the 5 pills (Figure 1B)[10] is determined for each volunteer.

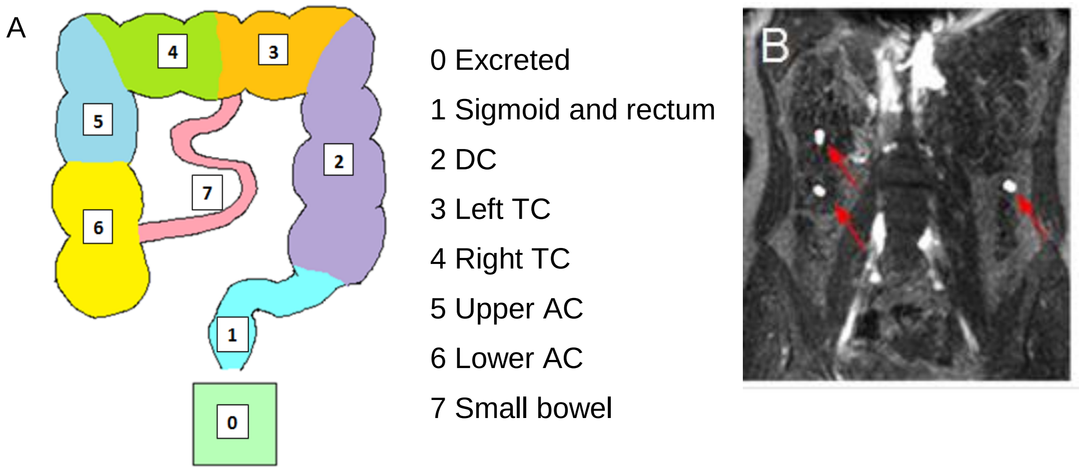

Figure 1: (**A**) Scoring system (**B)** an examples of maximum intensity from a volunteers T1-weighted TFE sequence scan 24 hours after swallowing 3 transit capsules showing the position of the capsules (indicated also by the red arrows) within the gut. On these sequences the capsules appear bright and in this case two can be seen in the ascending colon and 1 in the descending colon.

Colonic Morphology
Colonic volume will be calculated followed the method described recently by Pritchard et al.[8] Individual regional colon volumes will be manually segmented from the coronal data on each image slice using Analyze9™ software (Mayo Foundation, Rochester MN, USA). Each colon region will be identified within each coronal image slice, building a 3D representation of the morphology from which the volume of each region is measured. Information from axial data will be used to guide definition of the regions where anatomy is ambiguous.

Urinary Metabolomics

This will be done in the Centre for Analytical Biosciences at the School of Pharmacy, under the supervision of Prof David Barrett. Urine will be analysed using a validated direct infusion mass spectrometry method involving high resolution accurate mass analysis. This will enable the analysis of changes in levels of more than 35 compounds naturally found in urine including those linked with bacterial metabolism.

Stool

Stool samples will be stored until additional funding is confirmed. The intention will be to test stool microbiota using semi-quantitative PCR, using primers derived from a comprehensive study of the effects of OF on the microbiota[14]. Where this work involves NHS staff and premises a separate ethics submission will be made.

Stool will also be tested for short-chain fatty acid content. This will be done in the NDDC under the supervision of Dr Gulzar Singh using gas chromatography – mass spectrometry (GC-MS).

STATISTICS

Sample Size and justification

We will recruit up to 45 participants in order to gather 36 complete data sets.

Our pilot data found an 18% increase in colonic volume after normal diet was supplemented with OF. The null hypothesis would be that there is no change in colonic volume in either arm of the planned study (OF or maltodextrin). The effect of the low FODMAP diet is uncertain but should be the same in both arms. If our hypothesis is correct then volume may decrease in the maltodextrin arm.

Data from a previous 4-period crossover study of healthy volunteers[9] found a within-individual coefficient of variation for fasting colonic volume of 17% (unpublished). A sample size of 36, randomised 1:1, would give 80% power to detect a 15% increase in the change in volume between treatment arms ($\alpha = 0.05$, 1-tailed). Given our pilot work and the resulting study hypothesis we feel that a 1-tailed test is appropriate. Given the mechanistic nature of the study withdrawals and dropouts will be replaced.

Data will be assessed for normality using the Shapiro-Wilk test and other such methods as appropriate. Where normally distributed, endpoints will be assessed using parametric methods. The primary outcome measure will be analysed by t-test to compare the primary

endpoint between groups. Where the data is not normally distributed, non-parametric methods will be used.

Assessment of Efficacy

**Primary endpoint**	**Outcome Measure**
Percentage change in fasting colonic volume	Difference in mean response between arms
**Secondary endpoints**	**Outcome Measure**
Change in whole gut transit time	Difference between treatment arms by ranking, unless normally distributed
Percentage change in fasting colonic gas volume	Difference in mean response between arms
Change in fasting breath hydrogen	Difference in mean response between arms
Change in fasting breath methane	Difference in mean response between arms

GOOD CLINICAL PRACTICE

The study will be performed according to Good Clinical Practice principles. The volunteer study will undergo submission through the University of Nottingham Research Medical School Research Ethics Committee. All volunteers will give written informed consent. Their names will be entered into a subject log in the Trial Master File and a unique alpha numeric study code assigned. A simple Case Report Form will be kept for each volunteer.

ADVERSE EVENTS

We do not expect any adverse events following this intervention with a food supplement. Previous studies have used a greater OF dose without reporting adverse events[15].

Should a Serious Adverse Event occur it will be noted, dealt with according to SOPs and reported to the Ethics Committee.

REFERENCES

1. Gibson GR, Roberfroid MB. Dietary Modulation of the Human Colonic Microbiota: Introducing the Concept of Prebiotics. *Journal of Nutrition* 1995;125:1401-12.
2. Shepherd S, Parker F, Muir J, Gibson P. Dietary Triggers of Abdominal Symptoms in Patients With Irritable Bowel Syndrome: Randomized Placebo-Controlled Evidence. *Clinical Gastroenterology and Hepatology* 2008;6(7):765-71.
3. Staudacher HM, Whelan K, Irving PM, Lomer MCE. Comparison of symptom response following advice for a diet low in fermentable carbohydrates (FODMAPs) versus standard dietary advice in patients with irritable bowel syndrome. *Journal of Human Nutrition and Dietetics* 2011;24(5):487-95.
4. Halmos EP, Power VA, Shepherd SJ, Gibson PR, Muir JG. A diet low in FODMAPs reduces symptoms of irritable bowel syndrome. *Gastroenterology* 2014;146(1):67-75 e5.
5. Soret R, Chevalier J, De Coppet P, Poupeau G, Derkinderen P, Segain JP, et al. Short-chain fatty acids regulate the enteric neurons and control gastrointestinal motility in rats. *Gastroenterology* 2010;138(5):1772-82.
6. Kashyap PC, Marcobal A, Ursell LK, Larauche M, Duboc H, Earle KA, et al. Complex interactions among diet, gastrointestinal transit, and gut microbiota in humanized mice. *Gastroenterology* 2013;144(5):967-77.
7. Wu GD, Chen J, Hoffmann C, Bittinger K, Chen YY, Keilbaugh SA, et al. Linking long-term dietary patterns with gut microbial enterotypes. *Science* 2011;334(6052):105-8.
8. Pritchard SE, Marciani L, Garsed KC, Hoad CL, Thongborisute W, Roberts E, et al. Fasting and postprandial volumes of the undisturbed colon: normal values and changes in diarrhea-predominant irritable bowel syndrome measured using serial MRI. *Neurogastroenterol Motil* 2013.
9. Murray K, Wilkinson-Smith V, Hoad C, Costigan C, Cox E, Lam C, et al. Differential Effects of FODMAPs (Fermentable Oligo-, Di-, Mono-Saccharides and Polyols) on Small and Large Intestinal Contents in Healthy Subjects Shown by MRI. *Am J Gastroenterol* 2013.
10. Chaddock G, Lam C, Hoad CL, Costigan C, Cox EF, Placidi E, et al. Novel MRI tests of orocecal transit time and whole gut transit time: studies in normal subjects. *Neurogastroenterology & Motility* 2013.
11. Hoad CL, Cox EF, Gowland PA. Quantification of T(2) in the abdomen at 3.0 T using a T(2)-prepared balanced turbo field echo sequence. *Magnetic resonance in medicine : official journal of the Society of Magnetic Resonance in Medicine / Society of Magnetic Resonance in Medicine* 2010;63(2):356-64.
12. Placidi E, Marciani L, Hoad CL, Napolitano A, Garsed KC, Pritchard SE, et al. The effects of loperamide, or loperamide plus simethicone, on the distribution of gut water as assessed by MRI in a mannitol model of secretory diarrhoea. *Alimentary Pharmacology & Therapeutics* 2012;36(1):64-73.
13. Hoad CL, Garsed K, Marciani L, Cox EF, Costigan C, Spiller RC, et al. Measuring T1 of chyme in the ascending colon in health and diarrhoea predominant Irritable Bowel Syndrome. *Proceedings of the 20th Annual Meeting of the International Society for Magnetic Resonance in Medicine 2012* 2012:1275.
14. Dewulf EM, Cani PD, Claus SP, Fuentes S, Puylaert PG, Neyrinck AM, et al. Insight into the prebiotic concept: lessons from an exploratory, double blind intervention study with inulin-type fructans in obese women. *Gut* 2013;62(8):1112-21.
15. Gibson GR BE, Wang X, Cummings JH. Selective Stimulation of Bifdobacteria in the Human Colon by Oligofructose and Inulin. *Gastroenterology* 1995;108:975-82.
